# The Induction of G2/M Phase Cell Cycle Arrest and Apoptosis by the Chalcone Derivative 1C in Sensitive and Resistant Ovarian Cancer Cells Is Associated with ROS Generation

**DOI:** 10.3390/ijms25147541

**Published:** 2024-07-09

**Authors:** Šimon Salanci, Mária Vilková, Lola Martinez, Ladislav Mirossay, Radka Michalková, Ján Mojžiš

**Affiliations:** 1 Department of Pharmacology, Faculty of Medicine, Pavol Jozef Šafárik University, 040 01 Košice, Slovakia; simon.salanci@student.upjs.sk (Š.S.); ladislav.mirossay@upjs.sk (L.M.); radka.michalkova@upjs.sk (R.M.); 2 Institute of Chemistry, Faculty of Science, Pavol Jozef Šafárik University, 040 01 Košice, Slovakia; maria.vilkova@upjs.sk; 3 Flow Cytometry Unit, Biotechnology Programme, Spanish National Cancer Research Center (CNIO), 28029 Madrid, Spain; lmartinez@cnio.es

**Keywords:** ovarian cancer, chalcones, antiproliferative, oxidative stress, G2/M arrest, apoptosis, Nrf2, N-acetylcysteine

## Abstract

Ovarian cancer ranks among the most severe forms of cancer affecting the female reproductive organs, posing a significant clinical challenge primarily due to the development of resistance to conventional therapies. This study investigated the effects of the chalcone derivative 1C on sensitive (A2780) and cisplatin-resistant (A2780cis) ovarian cancer cell lines. Our findings revealed that 1C suppressed cell viability, induced cell cycle arrest at the G2/M phase, and triggered apoptosis in both cell lines. These effects are closely associated with generating reactive oxygen species (ROS). Mechanistically, 1C induced DNA damage, modulated the activity of p21, PCNA, and phosphorylation of Rb and Bad proteins, as well as cleaved PARP. Moreover, it modulated Akt, Erk1/2, and NF-κB signaling pathways. Interestingly, we observed differential effects of 1C on Nrf2 levels between sensitive and resistant cells. While 1C increased Nrf2 levels in sensitive cells after 12 h and decreased them after 48 h, the opposite effect was observed in resistant cells. Notably, most of these effects were suppressed by the potent antioxidant N-acetylcysteine (NAC), underscoring the crucial role of ROS in 1C-induced antiproliferative activity. Moreover, we suggest that modulation of Nrf2 levels can, at least partially, contribute to the antiproliferative effect of chalcone 1C.

## 1. Introduction

Ovarian cancer (OC) is one of the most lethal among gynecological malignancies. According to GLOBOCAN, there were more than 324,000 new cases of OC in 2022 alongside more than 206,000 deaths from this cancer [1]. Because it is often diagnosed at advanced stages, treatment options are limited and prognosis is poor. The standard approach to treating newly diagnosed advanced OC involves cytoreductive surgery followed by chemotherapy with carboplatin and paclitaxel [2]. Moreover, new therapeutic modalities, such as antiangiogenic therapy and PARP (poly-adenosine diphosphate (ADP)-ribose polymerase) inhibitors, have also been included in the OC treatment [3,4]. Despite advancements in treatment modalities, mortality of OC remains unacceptably high and the development of new, less toxic treatments for OC patients remains crucial.

In recent years, natural compounds have been intensively studied for their potential use in the prevention or therapy of human diseases, including cancers [5,6,7].

In the past decade, the number of studies focusing on the potential anticancer effects of chalcones, a precursor in the synthesis of flavonoids in plants, has increased. In addition to their numerous biological effects, another advantage is the simple structural scaffold of chalcones allowing their easy modification to create a variety of active analogs [8].

Chalcones, as was mentioned, have gained attention for their broad spectrum of biological actions such as anti-inflammatory, antidiabetic antimicrobial, cardioprotective, or antioxidant effects [9,10,11,12,13]. One of the rapidly developing areas in the study of the biological effects of chalcones is their chemopreventive and anticancer effect. For example, it has been documented chemopreventive effect of flavokawain A against bladder or prostate cancers [14,15] or licochalcone A and cardamonin in colon cancer [16,17]. Moreover, some chalcones, such as isoliquiritigenin, xantohumol, or flavokawain B also showed a chemotherapeutic effect as they significantly suppressed the growth of chemically or biologically induced tumors in experimental animals [18,19,20]. More information on the molecular aspects of the antiproliferative and anticancer effects of chalcones can be found in our recent review papers [21,22].

As stated above, chalcones have the potential to reduce the risk of oxidative stress. On the other hand, under certain conditions, they can also induce oxidative stress leading to cytotoxic effects in cancer cells [23,24,25]. Cancer cells, however, have developed a strategy to protect themselves against oxidative stress, where the activation of Nrf2 (nuclear factor erythroid 2-related factor 2) plays a key role in combating oxidative stress [26]. When cells are exposed to oxidative stress, Nrf2 is translocated into the nucleus and binds to specific DNA regions known as antioxidant response elements (ARE) [27]. Subsequently, the transcription of genes encoding antioxidant enzymes such as heme oxygenase-1 (HO-1), NAD(P)H: quinone oxidoreductase 1 (NQO1), glutathione S-transferases (GST), and many others is triggered [28]. Due to the protection of cancer cells against oxidative stress, Nrf2 is a potential target in cancer therapy [29,30].

Numerous studies indicate that chalcones are capable of both activating and inhibiting Nrf2 signaling.

Although most of them reported a protective effect of different chalcones against oxidative stress via Nrf2 activation [31,32,33,34], some chalcones have been reported to possess opposite action. Laphanuwat and co-workers (2022) documented the antiproliferative effect of Licochalcone A in cholangiocarcinoma cells associated with Nrf2 axis inhibition followed by oxidative stress induction [35]. Similarly, cardamonin, another natural chalcone, inhibited the growth of breast cancer cells, and this effect was associated with reduced Nrf2 protein expression and subsequent increased reactive oxygen species (ROS) accumulation [36]. Furthermore, as documented by Lim et al. (2013), chalcones via inhibition of the Nrf2-mediated defense mechanism can accelerate ROS production and enhance the sensitivity of cancer cells to anticancer drugs [37].

Recently, we found that chalcone-acridine hybrid 1C strongly suppressed the growth of colon cancer and melanoma cells, and this effect was mediated by pleiotropic mechanisms including induction of oxidative stress [38,39,40]. However, there is no information regarding the interaction of 1C with Nrf2. Therefore, the objective of this study was to investigate the mechanism of the antiproliferative effect of chalcone 1C in ovarian cancer cells focused on the modulation of Nrf2 signaling.

## 2. Results

### 2.1. MTT Screening Assay

**Figure 1:**
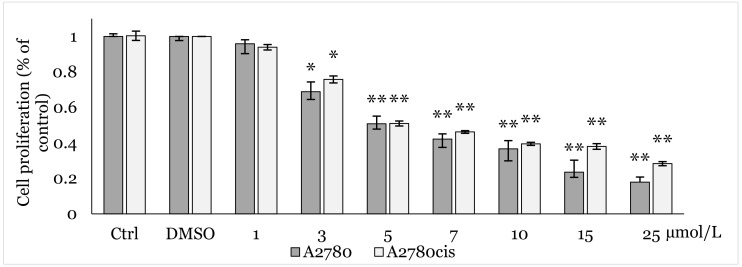
Antiproliferative effect of 1C on A2780 and A2780cis cells after 48 h exposure determined by MTT assay. Results show mean ± standard deviation calculated from three independent experiments. Statistical significance: * *p* < 0.05, ** *p* < 0.01 vs. control (DMSO).

The MTT colorimetric assay, performed to analyze the antiproliferative effect of 1C, showed a concentration-dependent cytotoxic effect with significant inhibition of cell viability at several concentrations starting from 3 μmol/L in both cell lines. Based on data obtained after 48 h exposure, the IC_50_ value was determined to be 6.59 ± 0.57 μmol/L in A2780 and 6.98 ± 1.12 μmol/L A2780cis cells. After additional testing, concentrations of 9 μmol/L and 7 μmol/L were used for the following experiments in A2780 and A2780cis cells respectively (Figure 1). The selectivity index against the non-tumor cells was not determined. It was previously determined to be 5.54 and 5.23 on the MCF-10A cells, with corresponding IC_50_ values of 36.54 ± 0.87 μmol/L by Gazdová, 2022 [40].

**Figure 2:**
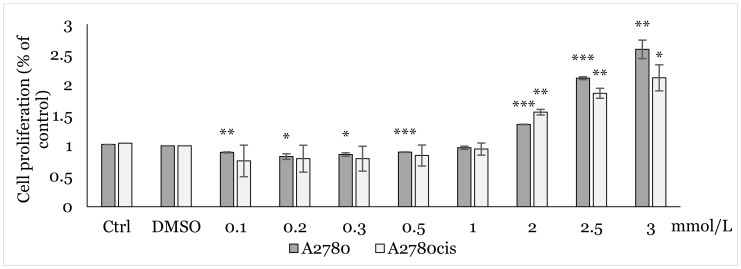
Effect of NAC on A2780 and A2780cis cells after 48 h exposure determined by MTT assay. Results show the mean ± standard deviation calculated from three independent experiments. Statistical significance: * *p* < 0.05, ** *p* < 0.01, *** *p* < 0.001 vs. control (culture medium).

**Figure 3:**
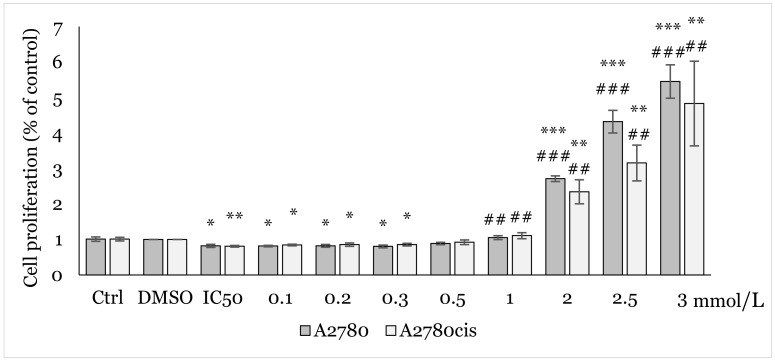
Effect of NAC/1C on A2780 and A2780cis cells viability after 12 h exposure determined by MTT assay. Results show the mean ± standard deviation calculated from three independent experiments. Statistical significance: * *p* < 0.05, ** *p* < 0.01, *** *p* < 0.001 vs. control (Ctrl) and ## *p* < 0.01, ### *p* < 0.001 vs. IC_50_ (1C).

**Figure 4:**
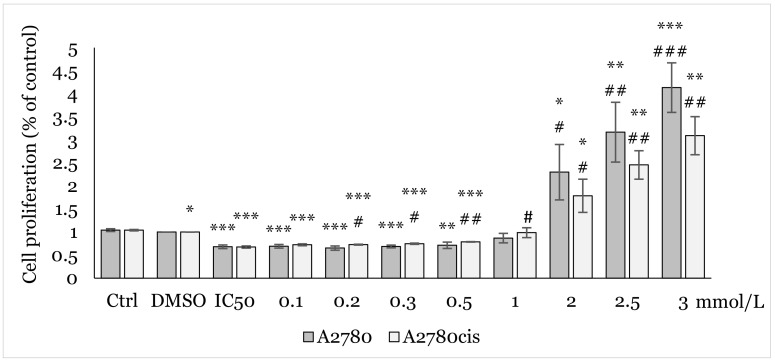
Effect of NAC/1C on A2780 and A2780cis cells viability after 24 h exposure determined by MTT assay. Results show the mean ± standard deviation calculated from three independent experiments. Statistical significance: * *p* < 0.05, ** *p* < 0.01, *** *p* < 0.001 vs. control (Ctrl) and # *p* < 0.05, ## *p* < 0.01, ### *p* < 0.001 vs. IC_50_ (1C).

**Figure 5:**
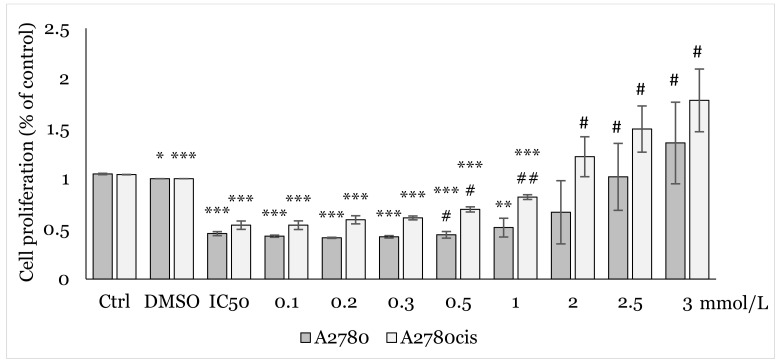
Effect of NAC/1C on A2780 and A2780cis cells viability after 48 h exposure determined by MTT assay. Results show the mean ± standard deviation calculated from three independent experiments. Statistical significance: * *p* < 0.05, ** *p* < 0.01, *** *p* < 0.001 vs. control (Ctrl) and # *p* < 0.05, ## *p* < 0.01, vs. IC_50_ (1C).

Using MTT assay, we analyzed the effect of various concentrations (0.1, 0.2, 0.3, 0.5, 1.0, 2.0, 2.5, 3.0 mmol/L) of NAC on cell proliferation. After 48h of exposure to NAC alone (Figure 2), and 12, 24, and 48 h exposures to combination NAC/1C, where the respective IC_50_ of 1C was used for each cell line (Figure 3, Figure 4 and Figure 5), a positive effect on cell viability was observed in both cell lines. The most significant protective effect was observed in the concentration range of 1.0–2.5 mmol/L compared to the control. The obtained results shown in Figure 2, Figure 3, Figure 4 and Figure 5 suggest that NAC positively contributed to cell survival by mitigating the cytotoxic effect induced by 1C. Based on the obtained data, the concentration of 2.5 mmol/L was determined as non-toxic and therefore used in subsequent experiments.

### 2.2. Effect of 1C and NAC on Oxidative Stress

**Figure 6:**
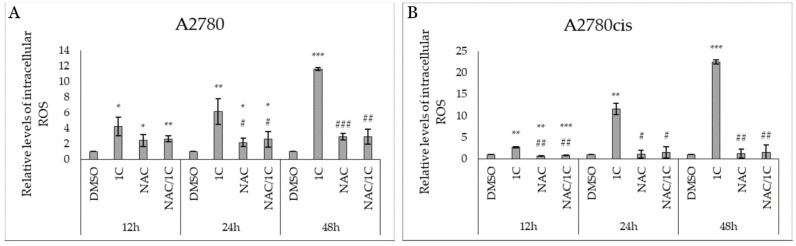
The influence of 1C, NAC, and their combination on ROS production in A2780 (**A**) and A2780cis (**B**) cells after 12, 24, and 48 h exposure. Data were obtained from three independent acquisitions. Statistical significance: * *p* < 0.05, ** *p* < 0.01, *** *p* < 0.001 vs. control (DMSO); # *p* < 0.05, ## *p* < 0.01, ### *p* < 0.001 vs. 1C.

To determine whether the cytotoxic effect of the tested substance 1C is mediated through oxidative stress, flow cytometric analyses focusing on reactive oxygen species (ROS) levels, superoxide anion, and lipid peroxidation were conducted. The results of ROS analysis (Figure 6A,B) revealed a significant increase in ROS production in both 1C-treated cell lines across all three incubation times compared to the control (DMSO). The highest elevation of ROS (*p* < 0.001 vs. DMSO) was observed after 48 h of exposure in both cell lines. Surprisingly, increased ROS production was also observed in the sensitive A2780 cells treated with the antioxidant NAC after 12 and 24 h of incubation. On the other hand, NAC did not stimulate ROS production in the resistant A2780cis cells. However, the combination of NAC/1C led to significant suppression in ROS production in A2780cis cells after 24 and 48 h of exposure and across all incubation periods in A2780cis compared to 1C-treated cells.

**Figure 7:**
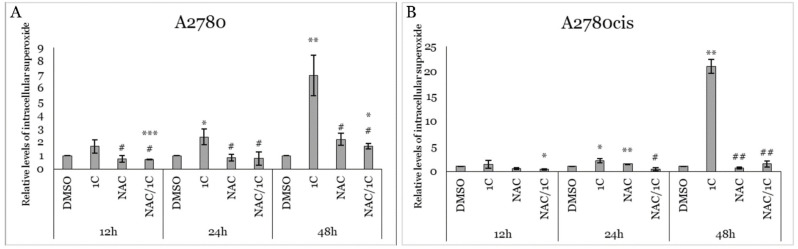
Effect on superoxide anion production in A2780 (**A**) and A2780cis (**B**) cells after 12, 24, and 48 h exposure to 1C, NAC, and combination NAC/1C. Presented data were obtained from three independent acquisitions. Statistical significance: * *p* < 0.05, ** *p* < 0.01, *** *p* < 0.001 vs. control (DMSO); # *p* < 0.05, ## *p* < 0.01 vs. 1C.

Next, we utilized MitoSOX™ Red to quantify mitochondrial superoxide anion (SO) levels in A2780 and A2780cis cells following 12, 24, and 48 h of incubation with 1C, NAC, and the NAC/1C combination. Our experiments revealed a significant increase in SO production after 24 and 48 h of incubation with 1C in both cell lines. As depicted in Figure 7A,B, the combination of NAC/1C significantly attenuated SO production in both cell lines after 24 and 48 h of incubation compared to treatment with 1C alone. However, this effect was less pronounced in sensitive cancer cells, as evidenced by significantly higher levels of SO in the NAC/1C group after 12 and 48 h of incubation compared to DMSO-treated cells.

**Figure 8:**
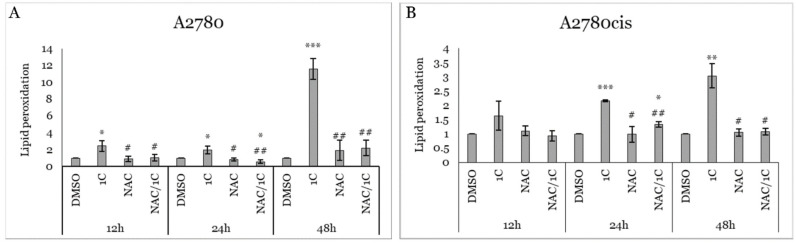
Impact of 1C, NAC, and their combination on lipid peroxidation in A2780 (**A**) and A2780cis (**B**) cells treated for 12, 24, and 48 h. Presented data were obtained from three independent acquisitions. Statistical significance: * *p* < 0.05, ** *p* < 0.01, *** *p* < 0.001 vs. control (DMSO); # *p* < 0.05, ## *p* < 0.01 vs. 1C.

Since free radicals significantly contribute to lipid oxidation and are closely associated with oxidative cell damage, we conducted further analysis on lipid peroxidation (LO) concentrations. Our results showed that 1C significantly increased LO levels in A2780 cells during all three incubation intervals, with the most notable effect observed after 48 h of incubation. Conversely, a significant increase in LO concentration in resistant A2780cis cells was observed only after 24 and 48 h of treatment. Additionally, no significant increase in LO concentration was noted in cells treated with NAC alone. However, NAC significantly suppressed LO production in 1C-treated cells after 12, 24, and 48 h of treatment in A2780 cells, and after 24 and 48 h of incubation in A2780cis cells (Figure 8A,B).

### 2.3. Cell Cycle Analysis

**Figure 9:**
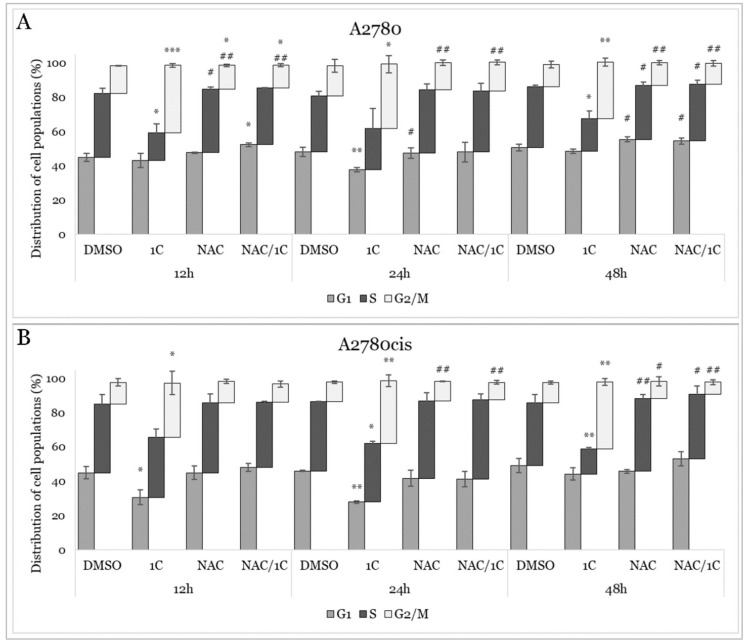
Analysis of cell cycle of A2780 (**A**) and A2780cis (**B**) cells affected by 12, 24, and 48 h lasting treatment with 1C, NAC and NAC/1C. Presented data were obtained from three independent acquisitions. Statistical significance: * *p* < 0.05, ** *p* < 0.01, *** *p* < 0.001 vs. control (DMSO); # *p* < 0.05, ## *p* < 0.01 vs. 1C.

To assess whether the decrease in viability induced by 1C was attributed to changes in cell cycle phases, flow cytometric analyses were conducted. As shown in Tables S1 and S2, chalcone 1C induced a significant G2/M arrest in both cell lines after 12, 24, and 48 h of treatment, accompanied by a decrease in the number of cells in the G1 or S phase (Figure 9A,B). The most pronounced effect was observed after 12 h of exposure in A2780 cells. Furthermore, to investigate the role of oxidative stress on the cell cycle, both cell lines were treated with the NAC/1C combination. Our results demonstrated that NAC prevented 1C-induced G2/M arrest in both cell lines across all time points studied.

### 2.4. Impact of 1C, NAC, and Their Combination on MMP

**Figure 10:**
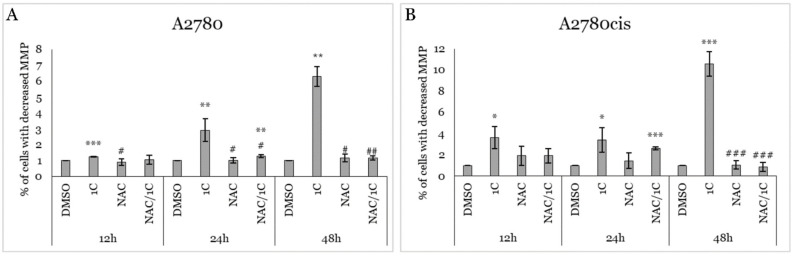
Effect of 1C, NAC, and their combination on the loss of mitochondrial membrane potential in A2780 (**A**) and A2780cis (**B**) cells after 12, 24, and 48 h exposure. Presented data were obtained from three independent acquisitions. Statistical significance: * *p* < 0.05, ** *p* < 0.01, *** *p* < 0.001 vs. control (DMSO); # *p* < 0.05, ## *p* < 0.01, ### *p* < 0.001 vs. 1C.

The mitochondrial membrane potential (MMP) is a critical electrochemical gradient across the inner mitochondrial membrane. It is essential for ATP production, regulation of metabolic processes, and overall cellular health. Moreover, there is a close association between MMP and superoxide production. A decrease in MMP often correlates with increased superoxide production, contributing to oxidative stress and cellular damage (Passos 2007). As mentioned above, treatment of ovarian cancer cells with 1C led to the overproduction of superoxide. This observation prompted us to analyze the impact of all treatments on MMP. Our experiment (Figure 10A,B) showed that exposure to 1C led to a significant increase in the number of cells with decreased MMP after 12, 24, and 48 h of incubation in both cell lines. Treatment with NAC in combination with 1C significantly reduced the number of MMP-decreased cells in the A2780 cell line after 12, 24, and 48 h compared to 1C alone. In contrast, in A2780cis cells, the protective effect of NAC was weaker and reached significance only after 48 h of incubation.

### 2.5. Apoptosis Detection

**Figure 11:**
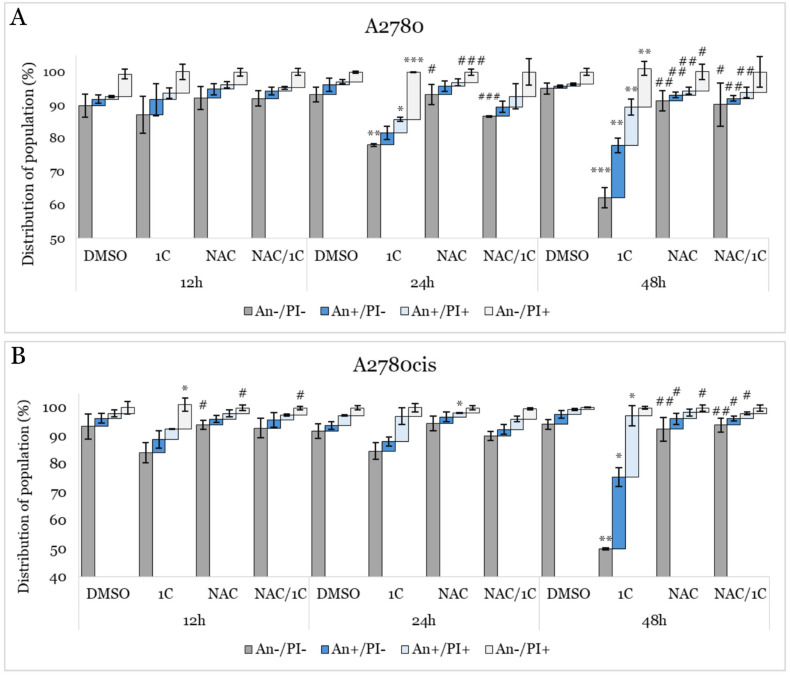
An/PI staining cytometric analysis of apoptosis influenced by 1C, NAC, and their combination in A2780 (upper (**A**)) and A2780cis (lower (**B**)) cells after 12, 24, and 48 h incubation. Presented data were obtained from three independent acquisitions. Statistical significance: * *p* < 0.05, ** *p* < 0.01, *** *p* < 0.001 vs. control (DMSO); # *p* < 0.05, ## *p* < 0.01, ### *p* < 0.001 vs. 1C.

A decrease in mitochondrial membrane potential is a critical event that can lead to apoptosis through the intrinsic pathway. One of the hallmark features of early apoptosis is the externalization of phosphatidylserine (PS) from the inner leaflet of the plasma membrane to the outer leaflet. Annexin V/propidium iodide (An/PI) double staining is a method used for detecting and distinguishing between viable (An−/PI−), early apoptotic (An+/PI−), late apoptotic (An+/PI+), and dead (An−/PI+) cells. As shown in Figure 11A,B and Table S3, our analysis demonstrated that 1C significantly reduced the population of viable A2780 cells after 24 and 48 h, and A2780cis cells after 48 h of exposure. Moreover, a considerable increase in early apoptotic and late apoptotic cells was observed in both cell lines. In A2780 cells, the population of dead cells increased after 24 and 48 h of exposure. Additionally, NAC significantly prevented 1C-induced apoptosis, as evidenced by the decrease in the number of cells in early and late apoptosis in both cell lines after 48 h of incubation, with a concurrent increase in viable cells compared to 1C alone.

#### 2.5.1. C modulates Expression of Cell Cycle and Apoptosis Regulating Proteins

Changes in the distribution of cell populations in the cell cycle and the associated induction of apoptosis are tightly regulated by changes in the phosphorylation or levels of regulatory proteins. This often occurs in response to DNA damage in an attempt to repair it.

**Figure 12:**
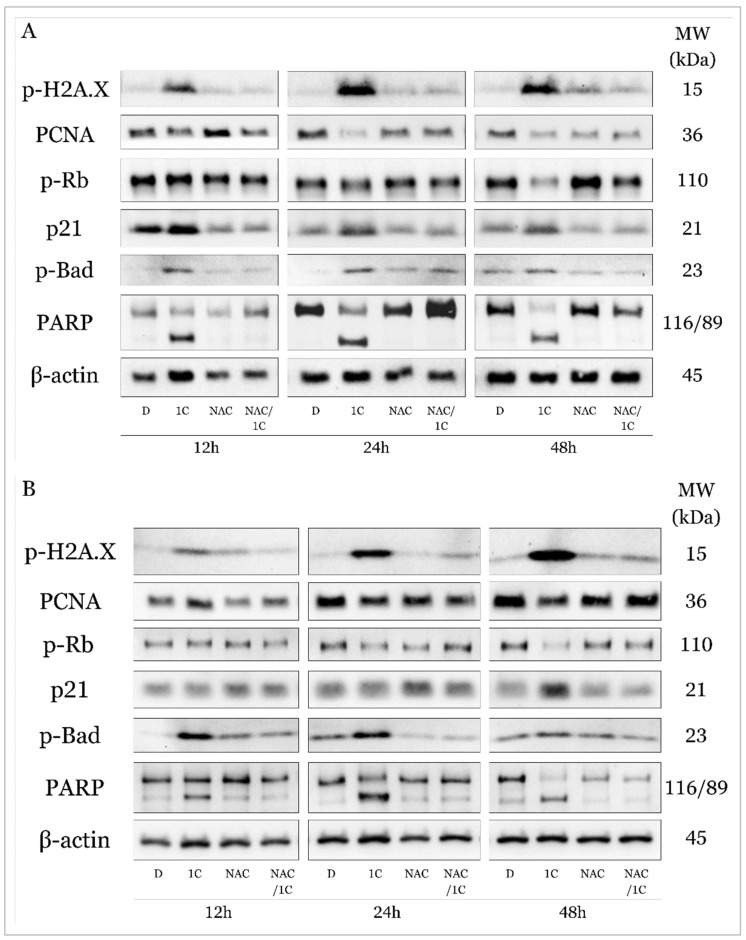
Western blot analysis of DNA damage, cell cycle, and apoptosis-associated proteins affected by 1C, NAC, and their combination in A2780 (**A**) and A2780cis (**B**) cells after 12, 24, and 48 h incubation. Representative figure.

**Figure 13:**
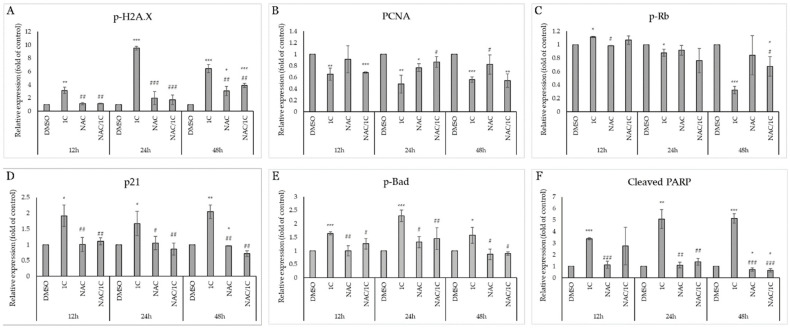
Densitometric analysis of phosphorylation of histone H2A.X (**A**), PCNA (**B**), phospho-Rb (**C**), p21 (**D**), phospho-Bad (**E**) and cleaved PARP (**F**) after 12, 24 and 48, and 72 h of 1C treatment in A2780 cells. Presented data were obtained from three independent acquisitions. Statistical significance: * *p* < 0.05, ** *p* < 0.01, *** *p* < 0.001 vs. control (DMSO); # *p* < 0.05, ## *p* < 0.01, ### *p* < 0.001 vs. 1C.

**Figure 14:**
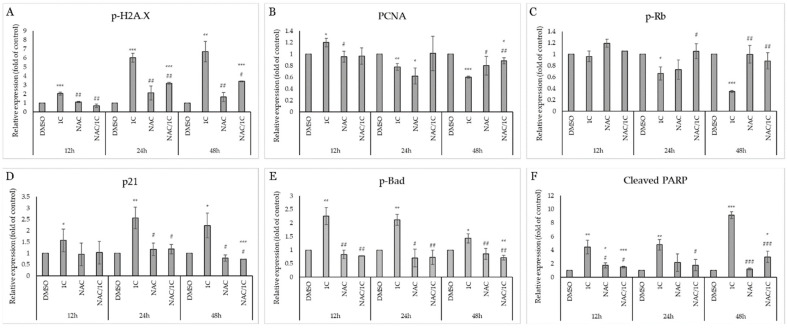
Densitometric analysis of phosphorylation of histone H2A.X (**A**), PCNA (**B**), phospho-Rb (**C**), p21 (**D**), phospho-Bad (**E**) and cleaved PARP (**F)** after 12, 24 and 48, and 72 h of 1C treatment in A2780cis cells. Presented data were obtained from three independent acquisitions. Statistical significance: * *p* < 0.05, ** *p* < 0.01, *** *p* < 0.001 vs. control (DMSO); # *p* < 0.05, ## *p* < 0.01, ### *p* < 0.001 vs. 1C.

Histone H2A.X is considered one of the basic markers of DNA damage, and it is essential in the repair of double-stranded DNA breaks and activates other kinases during DDR (DNA damage response). Western blot analysis showed that the studied chalcone 1C is able to increase the phosphorylation of H2A.X in both cisplatin-sensitive and resistant line A2780 at all exposure times (Figure 12A,B). This effect was significantly antagonized by the antioxidant NAC, which in connection with the abovementioned results (production of ROS, superoxide, lipid peroxidation, etc.) suggests that the effect of 1C is associated with oxidative damaged DNA (Figure 13A and Figure 14A).

PCNA (Proliferating cell nuclear antigen), which is also considered a marker of proliferation, plays a complex role in the process of repairing damaged DNA and its replication, proliferation, and transition of cells through the entire cell cycle. In A2780 cells, we observed a decrease in PCNA levels after only 12 h, and NAC had only 24 h of incubation in combination with changes in the expression of this protein (Figure 12A). In A2780cis cells, there was an increase in PCNA expression in cells exposed to 1C after 12 h; after 24 and 48 h, this effect was significantly reversed, and after 48 h NAC increased PCNA expression compared to 1C alone (Figure 12B and Figure 14B).

PCNA interacts with the transcription factor E2F, the most important regulator of which is the Rb tumor suppressor protein. Phosphorylation of Rb means the loss of its ability to prevent the interaction of E2F with the entire transcription machinery and thus facilitate the G1/S transition. As shown in Figure 12, in both tumor cell lines A2780 and A2780cis, under the influence of 1C, a significant downregulation of phosphorylated Rb occurred after 24 h; after 48 h of exposure in combination with NAC, this effect was suppressed (Figure 13C and Figure 14C). These results are complementary to the cell cycle analysis, which confirmed a cell cycle block in the G2/M phase (Figure 10A,B), which disappeared after exposure to the NAC/1C combination.

Among the most important tumor suppressors is the protein p21 Waf1/Cip1, which is considered a promiscuous inhibitor of all cyclin complexes and cyclin-dependent kinases and is capable of binding and inhibiting PCNA, thereby significantly contributing to stopping cell cycle progression. Our results showed that p21 was significantly upregulated in A2780 and A2780cis cells at all exposure times (from 12 to 48h), but NAC was able to suppress this effect and p21 levels were at the control level in the cisplatin-sensitive line at 24h, in A2780cis already after 12 h in cells incubated with NAC and subsequently 1C (Figure 12A,B, Figure 13D and Figure 14D).

All these processes, if the damaged DNA is not repaired and the cells are not able to restore the mechanisms ensuring the continuation of the cell cycle, can lead to the induction of cell death. As shown in Figure 12, the studied substance also significantly affected proteins considered as markers of apoptosis.

The pro-apoptotic protein Bad belongs to the Bcl-2 family of proteins, whose role is to prevent the interaction of anti-apoptotic proteins from the Bcl-2 family and other pro-apoptotic proteins and thus ensure the induction of mitochondrial (internal apoptosis pathway). Our experiments demonstrated a significant effect of 1C on the phosphorylation of this protein in both ovarian tumor lines at all times of incubation with chalcone, the effect of which was antagonized by the antioxidant NAC (Figure 12A,B, Figure 13E and Figure 14E).

Upon induction of the intrinsic or extrinsic pathway of apoptosis, activation of the caspase cascade results in the cleavage of intracellular molecules, including such as PARP (poly (ADP-ribose) polymerase), which is involved in the repair of damaged DNA in response to environmental stress. If it is cleaved by caspases, DNA damage is irreversible, and therefore cleaved PARP serves as a marker of ongoing apoptosis. Similar to the results from the analysis using Annexin V/PI staining proving apoptosis, the protein analysis showed a significant increase in the level of cleaved PARP in cells exposed to 1C already after 12 h of exposure (Figure 12A,B). As was expected, NAC also reduced or completely suppressed the pro-apoptotic effect of chalcone in this case (Figure 13F and Figure 14F).

These results indicate that chalcone 1C has a direct or indirect proapoptotic effect on both cisplatin-sensitive and cisplatin-resistant human ovarian carcinoma cells A2780 and A2780cis.

#### 2.5.2. 1C Modulates Signaling Pathways Associated with Oxidative Stress

Signaling pathways associated with ROS production and response to oxidative stress may represent a mechanism that significantly contributes to the initiation, promotion, and progression of carcinogenesis, but on the other hand, may lead to cell death.

**Figure 15:**
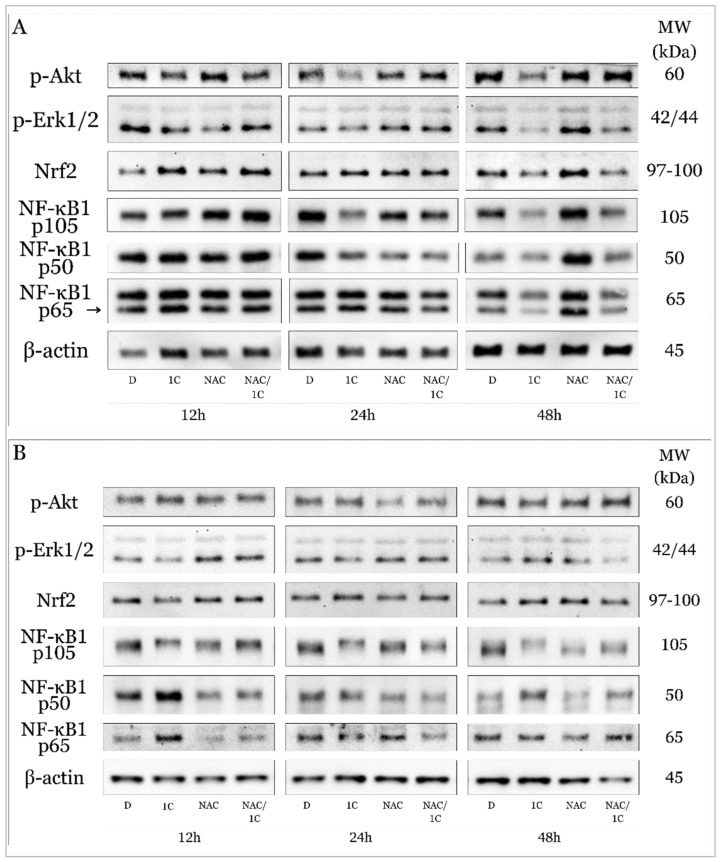
Western blot analysis of oxidative stress response associated proteins affected by 1C, NAC, and their combination in A2780 (**A**) and A2780cis (**B**) cells after 12, 24, and 48 h incubation. Representative figure.

**Figure 16:**
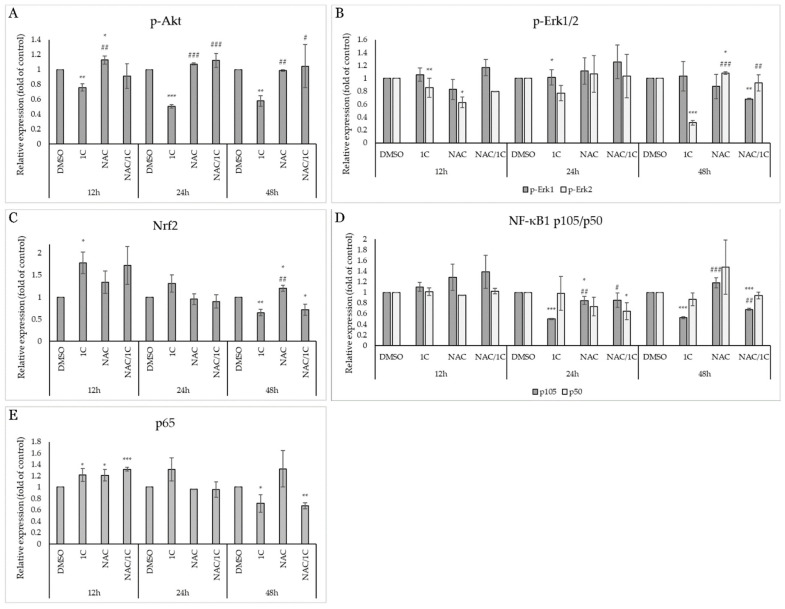
Densitometric analysis of phosphorylation of Akt (**A**), phospho-Erk1/2 (**B**), Nrf2 (**C**), NF-κB1 105/p50 (**D**) and p65 (**E**) after 12, 24 and 48, and 72 h of 1C treatment in A2780 cells. Presented data were obtained from three independent acquisitions. Statistical significance: * *p* < 0.05, ** *p* < 0.01, *** *p* < 0.001 vs. control (DMSO); # *p* < 0.05, ## *p* < 0.01, ### *p* < 0.001 vs. 1C.

**Figure 17:**
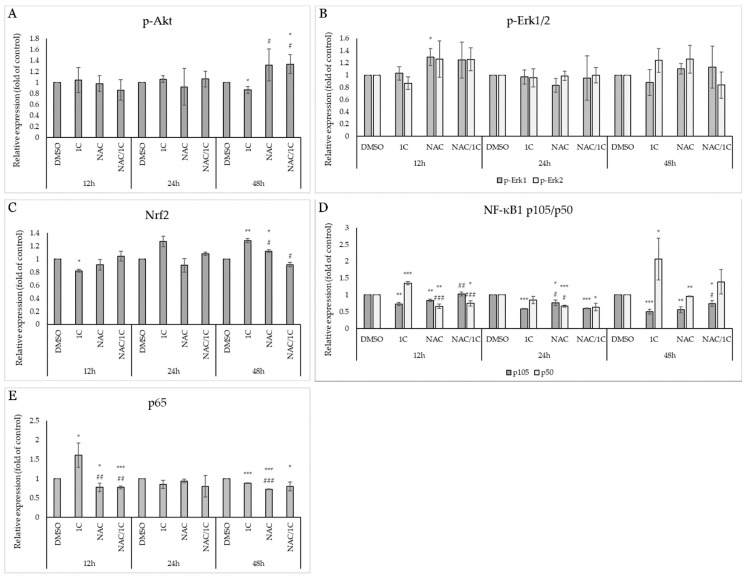
Densitometric analysis of phosphorylation of Akt (**A**), phospho-Erk1/2 (**B**), Nrf2 (**C**), NF-κB1 105/p50 (**D**), and p65 (**E**) after 12, 24 and 48, and 72 h of 1C treatment in A2780cis cells. Presented data were obtained from three independent acquisitions. Statistical significance: * *p* < 0.05, ** *p* < 0.01, *** *p* < 0.001 vs. control (DMSO); # *p* < 0.05, ## *p* < 0.01, ### *p* < 0.001 vs. 1C.

Also known as protein kinase B, Akt plays a critical role in controlling cell proliferation and cell survival as well as cell death. Its functions include interactions with proteins from the Bcl-2 family, it is involved in the regulation of the cell cycle towards its progression, metabolism, and autophagy and its abnormal overexpression or activation of AKT has been observed in many cancers. Western blot analysis demonstrated that chalcone 1C treatment induced a significant downregulation of phosphorylated Akt in cisplatin-sensitive A2780 cells, which was antagonized in combination with NAC (Figure 15A and Figure 16A). Interestingly, we did not observe such a significant effect in cisplatin-resistant A2780cis cells, where there was only a slight decrease in the level of phospho-Akt after 48 h of exposure, and on the other hand, NAC increased Akt phosphorylation after 48 h. Their combination produced no effect (Figure 15B and Figure 17A).

ROS is also able to activate signaling pathways associated with MAPKs (Mitogen-activated protein kinases). It is known that the activation of Erk1/2 generally promotes cell proliferation and ensures cell survival, and therefore it is considered a potential therapeutic target for the treatment of some tumors. On the other hand, Erk1/2 can be activated in response to DNA damage and thus be involved in the induction of apoptosis and thus exhibit a pro-apoptotic effect. In our study, we observed a significant decrease in Erk2 phosphorylation in A2780 cells after exposure to 1C with a maximum at 48 h (Figure 15A and Figure 16B). No changes in Erk1/2 phosphorylation status were noted in the resistant line A2780cis (Figure 15B and Figure 17B).

Proteins that significantly regulate the response to oxidative stress include Nrf2, whose role is to control the cellular oxidant level and trigger the transcription of proteins that regulate antioxidant systems in the cell in an attempt to eliminate and resist oxidative stress. In the cell, its levels are regulated by a direct inhibitor ensuring its ubiquitination, the Keap1 protein. Our analyses showed that in the non-resistant line A2780, there was an initial increase in Nrf2 levels (after 12 h), but after 48 h of exposure, its level significantly decreased both alone and in combination with NAC (Figure 15A and Figure 16C). Surprisingly, in the cells of the resistant line, we observed an initial decrease in the expression of Nrf2 (after 12 h of exposure), but after 48 h of incubation at 1C, there was a significant increase in the levels of this protein (Figure 15B and Figure 17C).

The family of transcription factors of the nuclear factor κB plays a crucial role in the rapid response to inflammation and the immune response. Factors that induce its activation include ROS, ionizing radiation, cytokines, etc. As activated transcription factors, they are known to induce genes responsible for proliferation and cell survival and thus help cells avoid apoptotic cell death. Since they can promote tumorigenesis, they have a suitable potential for antitumor treatment research. Our experiments show that in the sensitive line A2780 there was a decrease in the expression of precursor p105 without a simultaneous increase in the levels of the active form of NF-κB1 p50. This effect was significantly antagonized in cells exposed to NAC and the NAC/1C combination, especially after 48h (Figure 15 and Figure 16D). On the other hand, after 48 h of incubation with 1C, in addition to the reduction of p105, we observed a significant upregulation of the active form of p50 in A2780cis (Figure 15A,B and Figure 17D). The NF-κB family also includes the protein p65, also known as RelA, which, like the transcription factors mentioned above, contributes to the proliferation and migration of tumor cells. In this case, in both lines A2780 and A2780cis, p65 upregulation was observed after 12 h of chalcone treatment, but NAC alone and in combination with 1C had the opposite, line-dependent effect. After 48 h exposure, 1C induced a significant decrease in p65 levels in both lines (Figure 15A,B, Figure 16E and Figure 17E).

## 3. Discussion

Despite constant research in the field of oncology, the advent of ever more accurate diagnostics, and targeted treatment, cisplatin (DPP) remains the first-line treatment for ovarian cancers. Although the majority of patients respond well to treatment and this treatment is considered effective, patients treated with DPP may develop resistance through various mechanisms, ultimately leading to treatment failure [41]. This problem leads to the use of new approaches and strategies for the treatment of ovarian malignancies, namely the use of a combination of other, non-platinum drugs to which these tumors may be sensitive, nanoparticle delivery systems, immunotherapy as well as phytochemicals, which could act as separate drugs or chemosensitizers [42].

Several studies have been published focusing on chemoresistance and the potential use of natural chalcones as modulators of resistance-related signaling pathways and processes such as autophagy, Wnt/β-catenin, ABC transporters, and others [43,44,45,46]. In our study, we focused on the role of oxidative stress and the potential mechanism of action of the chalcone 1C ((2E)-3-(acridin-9-yl)-1-(2,6-dimethoxyphenyl)prop-2-en-1-one) on human ovarian carcinoma tumor cells A2780 and the cisplatin-resistant line A2780cis. Differences in the effect of cisplatin were recently published in our recent study where the IC_50_ for the sensitive line exposed to cisplatin after 72 h was 1.64 ± 0.35 μmol/L and for the resistant A2780cis cells the IC_50_ was 12.73 ± 2.55 μmol/L [47]. The studied acridine derivative of chalcones 1C had a high selectivity index (5.54 and 5.23) against healthy human epithelial breast cells MCF-10A [40].

That chalcones are potentially similarly effective on resistant cells was also proven by the study of Jung et al. [48], who compared the effectiveness of 2′, 3′, 5-trimethoxychalcone (DPP23) on ovarian tumor cells A2780 and A2780/CisR at a concentration of 10 μmol/L over different periods, showing a maximum effect after 24 h. Similarly, we also monitored the involvement of ROS in a possible antiproliferative effect in our experiments. We recorded a significant generation of ROS after 12 h of incubation with 1C in both lines. For the experiments, we selected a concentration of 2.5 mmol/L N-acetylcysteine (NAC) as an antioxidant that could potentially antagonize oxidative stress induced by 1C [39]. Similar to other authors, we monitored the time-dependent effect of the studied compound on proliferation and viability, which decreased with the length of exposure [49]. On the other hand, NAC counteracted the anti-proliferative and pro-apoptotic effects and thus increased the viability at each exposure time (12 to 48 h). Similar results, along with an increase in intracellular ROS levels, were also reported by others who studied isobavachalcone on HL-60 cells (acute myeloid leukemia), flavokawain B on human melanoma cells (A375, A2058) or the synthetic chalcone derivative S17 on human gastric cancer cells (MCG803, HGC27, and SGC7901) [50,51,52].

ROS are naturally produced as products of oxygen metabolism, with main sources including mitochondria, as well as the endoplasmic reticulum and peroxisomes. Elevated ROS levels can arrest tumor growth by persistently inhibiting the cell cycle and induce apoptosis through either extrinsic or intrinsic pathways [53]. The main source of ROS is mitochondrial superoxide, which further generates secondary ROS species and serves as a recognized mediator of apoptosis. Several natural substances with potential antitumor and chemopreventive effects were found to act through a similar mechanism, often linked to endoplasmic reticulum stress and the initiation of the extrinsic pathway of apoptosis [54,55]. We observed an increase in the superoxide radical from 24 h of incubation with chalcone 1C, whose effect, similar to that of ROS, disappeared after incubation with the ROS scavenger NAC. Similar findings have been reported on A2780 ovarian carcinoma, hepatocellular carcinoma, and others exposed to synthetic chalcones or their complexes with heavy metals [56,57]. In addition, we also noted a significant increase in the population of cells showing lipid peroxidation in cisplatin-sensitive cells after 12 h and in resistant cells after 24 h of incubation with 1C. Also, in this case, the effect was antagonized by the antioxidant capacity of NAC. Moreover, studies suggest that the induction of lipid peroxidation following chalcone action, along with ROS generation and intracellular iron accumulation, can serve as a marker of ongoing ferroptosis [58,59]. Su et al. (2019) clarify the direct relationship between the production of superoxide anion, various ROS, lipid peroxidation, and damage to DNA, proteins, lipids, and even membranes and whole organelles. Polyunsaturated fatty acids containing phospholipids can generate alkoxyl (RO·) radicals. These products of lipid peroxidation, after exhaustion of anti-oxidation systems, induce apoptosis, autophagy, and other types of cell death via different signaling pathways [60].

Since oxidative stress leads to DNA damage and genomic instability, cells in an attempt to repair the resulting damage can activate repair systems, block the cell cycle, and, in case of irreparable damage, induce cell death [61]. Flow cytometric analysis showed that 1C is capable of inducing cell cycle arrest in the G2/M phase, while this block was not observed after incubation with NAC or with the combination of NAC/1C. This effect may be related to changes in the levels of DNA damage markers and cell cycle regulatory proteins. In addition, it was found that mitotic arrest itself further enhances ROS and oxidative damage to proteins and nucleotides [62]. Similar to our experiments, other authors also observed changes in the levels of histone H2A.X as an important biomarker of DNA damage, an increase in the level of p21, and a cell cycle arrest in G2/M after exposure to melanoma cells SK-MEL-5 and SK-MEL-28 to a new methoxy derivative of chalcones. Consistent with our study, NAC reduced the levels of these proteins to control levels and reversed cell cycle arrest [63].

After the response to DNA damage and the involvement of p21 and other important tumor suppressors, cyclins, cyclin-dependent kinases, and transcription factors regulating cell proliferation are affected [64]. We recently demonstrated the ability of chalcones to change the levels of these proteins in a model of human breast tumor cells (MDA-MB-231, MCF-7), where the chalcone ZK-CH-11d significantly reduced the phosphorylation of the Rb protein, which is inhibited in the phosphorylated state [65]. Reducing its phosphorylation increases its tumor suppressor activity against the transcription factor E2F, which positively regulates cell cycle progression [66]. We therefore consider the reduction of Rb phosphorylation in both A2780 and A2780cis tumor lines after exposure to 1C to be one of the contributing factors by which this chalcone arrests the cell cycle. Proliferating cell nuclear antigen (PCNA) is among the factors affecting cell cycle, DNA damage repair, replication, chromatin assembly, and many other essential processes [67]. In our experiments on cisplatin-resistant and sensitive ovarian cancer lines, we observed that after exposure to chalcone, the level of this proliferation marker decreased in the A2780 line after 12 h. In the resistant line A2780cis, first upregulation occurred (after 12 h incubation), but after 24 and 48 h, PCNA was significantly downregulated. We assume that this phenomenon is related to the effort of resistant cells to continue to proliferate and repair potential DNA damage. PCNA reduction with simultaneous upregulation of tumor suppressors such as p21, p27, and others was observed after treatment with natural and synthetic chalcones on human prostate (PC3), bladder (T24), and colorectal cancer (HCT116) tumor cells [68,69,70].

If cell cycle arrest is not sufficient to repair damaged DNA, the cell undergoes apoptotic cell death. In the presence of functional p21 proteins, a decrease in anti-apoptotic phospho-Rb, and others, the cell induces the expression of proapoptotic factors, which include BH3 domain-only proapoptotic proteins (Puma, Noxa, Bad, Bax, Bak, p53); death receptors (Fas, DR4, DR5); and apoptosis execution factors (Apaf1, caspases) [71]. Flow cytometric analysis of apoptosis using Annexin V/PI double staining demonstrated that the induction of apoptosis is directly related to oxidative stress induced by 1C. Cells of the cisplatin-sensitive line A2780 showed signs of apoptosis (An+/PI−, An+/PI+) and necrosis (An-/PI+) after 24 h. In the case of the resistant line A2780cis, we recorded positivity for these markers only after 48 h of incubation. We believe that this phenomenon is also related to the resistance mechanisms of this line. NAC attenuated or reduced these effects to the level of control cells. A study by Qi et al. (2014) presented results on three tumor human ovarian lines (A2780, A2780/CDDP, and SKOV3), where they evaluated the effect of the tetramethoxychalcone derivative and found that it was able to reduce tumor cell viability in a time- and dose-dependent manner. Even in this case, the resistant line required a higher concentration to reach the half-inhibitory concentration [72]. Natural flavokawan B showed a similar effect on melanoma cells A375 and A2058 [73].

We assume that the pro-apoptotic effect induced by 1C was associated with the activation of the intrinsic pathway of apoptosis, which is mediated by the interaction of pro-apoptotic members of the Bcl-2 family of proteins with mitochondria. The involvement of the mitochondrial pathway of apoptosis is evidenced by a change in the levels of phosphorylated Bad, which indicates an imbalance in the ratio of Bcl-2 proteins, resulting in a significant increase in the population of cells with reduced mitochondrial potential (MMP). Mitochondrial outer membrane permeabilization (MOMP) initiates the activation of the caspase cascade [74]. The result of this machinery is irreversible damage to intracellular contents and irreparable damage to DNA. Therefore, cleavage of PARP (poly(ADP-ribose) polymerase-1) is considered an important marker of apoptosis. Western blot analysis of proteins showed that 1C effectively induces PARP cleavage, yet NAC is able to reverse the effect of 1C in both tumor lines at all exposure times. Many studies investigating the antiproliferative and potentially antitumor potential of chalcones describe similar results, including involvement of Bcl-2 proteins, changes in MMPs, cytochrome c release, induction of caspase-activating cleavage, and irreversible PARP cleavage [75,76,77,78].

Although it is clear that 1C has a pro-apoptotic effect and induces cell cycle arrest, to further clarify its effect, we studied some signaling pathways involved in the regulation of intracellular processes associated with oxidative stress. These undoubtedly include MAPK signaling, members of the NF-κB family, or the Keap1/Nrf2 pathway [61]. Akt, also known as protein kinase B, activated in several tumors is closely related to the maintenance of metabolic homeostasis. Hyperactivated PI3K/Akt/mTOR signaling was found in ovarian carcinomas [79]. In these types of tumors with hyperactivated Akt, ROS production can sensitize cells to ROS-induced apoptosis [80]. Our results point to the fact that chalcone was able to suppress Akt phosphorylation in cisplatin-sensitive A2780 cells as early as 12 h of incubation. The ability to suppress tumor cell growth and induce apoptosis associated with the inhibition of Akt phosphorylation has also been documented in other natural and synthetic chalcones. Deeb and co-authors [81] documented the proapoptotic effect of xanthohumol, while Wani and co-authors [82] found that a new quinazolinone chalcone derivative significantly inhibited Akt phosphorylation and induced cell death in colorectal tumors. On the other hand, our results showed that in resistant cells chalcone 1C suppressed Akt phosphorylation only after 48 h. We believe that the differences in effect between cell lines may be related to cisplatin resistance [83]. It is known that cisplatin-resistant cells often have hyperactivated Akt, which leads to reduced sensitivity to apoptotic signals, increased activity of DNA repair mechanisms, and activation of survival signals, allowing cancer cells to evade cisplatin-induced apoptosis [83,84,85].

Lee et al. (2024) [86] studied the effect of licochalcone C on sensitive and oxaliplatin-resistant HCT116 colorectal cancer cells and their findings were similar to the results of our experiments. Chalcone caused a massive downregulation of phosphorylated Akt in both lines, and the decrease in cell viability was also significantly attenuated after combination with NAC where the resistant cell line was less sensitive to the LCC effect in a dose-dependent manner.

An extremely interesting result is the changes in the phosphorylation of phospho-Erk1/2, where we found that while in the A2780 line, there was a significant downregulation of the phosphorylation of this protein after 48 h of exposure to 1C, we did not detect any effect on the phosphorylation of this protein in the resistant line A2780cis. Cisplatin treatment has been found to activate ERK in ovarian cancer cells and ERK activation protects ovarian cancer cells from cisplatin-induced death [87]. The studied chalcone was not able to suppress this member of MAPKs. However, the effect of chalcones on Erk1/2 phosphorylation has been described in many studies [88,89,90].

Similarly, interesting is the response of cells to 1C treatment and changes in Nrf2 expression, which were cell-dependent. As described in the introduction, chalcones can have the opposite effect on Nrf2 expression. In our experiments, we found that 1C downregulated this transcription factor in A2780 cells, but its expression was increased in resistant A2780cis cells. Reducing Nrf2 activity may help sensitize cells to anti-tumor therapy. Although increased Nrf2 activity protects both healthy and malignant cells against oxidative stress by activating antioxidant mechanisms [91], this fact had no effect on the inhibition of proliferation and apoptosis induced by 1C. It is thought that changes in Nrf2 levels may be related to changes in the metabolism of cisplatin-resistant tumor cells. Since Nrf2 exerts a significant effect on the antioxidant defense of cells against oxidizing agents by dissociating from binding with its negative regulator Keap, accumulating in the nucleus, binding to ARE (antioxidant response element), and inducing the expression of its target genes involved in the antioxidant response, detoxification, metabolism, and inflammation such as HO-1, GSGs, NQO1 and others [92]. Some hypothesize that cysteine may be a key molecule that has a direct effect on viability, and its presence may be a survival mechanism to protect cells from mitochondrial death induced by oxidative stress [93]. Whether 1C induced changes in expression, affected nuclear translocation, or interaction with its natural inhibitor Keap1 is the subject of our further investigation. However, several authors have described a decrease in the levels of cytoplasmic Nrf2 and its increase in the nucleus of both tumor and healthy cells [94,95]. Studying the action of chalcones on both cisplatin-sensitive and -resistant cells may better explain their potential use as chemosensitizers in the treatment of tumors [96].

Key regulators of cell survival include transcription factors from the NF-κB family, whose aberrant activation has been found in many diseases, including malignancies [97]. In our study, we observed changes in the expression of p105 as a precursor, p50 as an active form of NF-κB and p65, which forms a complex with p50, which is the best-described heterodimer of this family so far and controls the transcription of various genes in response to stress. This complex is activated by a variety of stimuli that include growth factors, cytokines, such as TNF and IL-1, LPS, UV, pharmacological agents, and stress [98]. In connection with our experiments, we again noted cell-dependent differences between sensitive and cisplatin-resistant lines of human ovarian carcinoma. In the A2780 line, there was a time-dependent decrease in the expression of both p105 and p65, in A2780cis an imbalance between the individual proteins was observed. The level of p105 as a precursor decreased, but the level of the active form increased significantly. The p65 protein increased after 12 h of incubation but significantly decreased after 48 h. It follows that the effect of chalcone 1C is cell-dependent as well as time-dependent. Papierska et al. [99] noted an interesting variability between the chalcones themselves and their effect on Nrf2 signaling, NF-κB, and the STAT3 pathway. However, most authors describe the anti-inflammatory and inhibitory effects of chalcones on this pathway [17,35,100]; even in SKOV3 ovarian cancer cells or MDA-MB-231 (triple-negative breast carcinoma), a reduced DNA binding activity induced by LPS was described [101]. The antagonistic effect of NAC on the action of isoliquiritin as a modulator of the MAPK signaling pathways, NF-κB, and the cell cycle was described in an extensive study conducted by Wang (2019) [102]. Similar to what we mentioned above, the levels of NF-κB family members are extremely sensitive to changes induced by extracellular influences such as anticancer agents, UV, and LPS. We assume that resistance to cisplatin may be responsible for more significant changes in p50/105 levels in the A2780cis cell line. activated NF-κB has been identified as a key mechanism of cisplatin resistance. In our experiments, we found that 1C was able to modulate this signaling already after 12 h of exposure in the resistant line, but only after 24 h in the sensitive line [103]. Metabolic reprogramming, autophagy, and a complex network of connections with other pathways such as Nrf2, JNK, JAK/STAT3, PTEN/mTOR, necroptosis, and many others are also related to Nf-κB signaling and chemoresistance; therefore, due to the antitumor potential of chalcone 1C and its effect on chemoresistance, it is necessary to carry out further experiments using inhibitors and other modulators of these events [97].

In our study, we pointed out the ability of chalcone 1C to inhibit the growth and proliferation of cisplatin-sensitive and cisplatin-resistant human ovarian carcinoma cells A2780 and A2780cis, which was associated with oxidative stress. We noted a significant increase in apoptotic markers as well as cell cycle arrest. All of these findings were correlated with increased ROS accumulation, increased intracellular superoxide anion, and lipid peroxidation, all of which were suppressed by the administration of the antioxidant NAC. 1C also significantly affected the signaling pathways involved in the response to oxidative stress, especially in the cisplatin-sensitive line. It is our further goal to clarify these differences and thus contribute to a better understanding of the mechanisms contributing to resistance to conventional treatment of ovarian cancer.

## 4. Materials and Methods

### 4.1. Tested Compounds

The chalcone analog (2E)-3-(acridin-9-yl)-1-(2,6-dimethoxyphenyl)prop-2-en-1-one (1C) was synthesized by Mária Vilková at the Faculty of Science, P.J. Šafárik University, Košice. The structure of the compound was confirmed using 1H and 13C NMR, IR spectroscopy, and mass spectrometry. The studied compound was dissolved in DMSO (Sigma-Aldrich, St. Louis, MO, USA) at a final, non-toxic concentration of 0.05%.

### 4.2. Cell Culture

The cell lines A2780 (human ovarian adenocarcinoma) and A2780cis (human ovarian adenocarcinoma cisplatin-resistant cell line (ATCC, Manassas, VA, USA) were cultured using RPMI 1640 growth medium (Biosera, Kansas City, MO, USA). The culture medium was supplemented with 10% fetal bovine serum (FBS) (Invitrogen, Carlsbad, CA, USA), and 1× HyCloneTM Antibiotic/Antimycotic Solution (GE Healthcare, Piscataway, NJ, USA). Standard conditions with an atmosphere containing 5% CO_2_ at 37 °C were maintained during cultivation. Cell viability, determined by trypan blue exclusion before each experiment, was consistently greater than 95% before each experiment.

### 4.3. MTT Viability Assay

For the initial antiproliferative activity analysis and determination of the IC_50_ of the tested compounds, the MTT (3-(4,5-dimethylthiazol-2-yl)-2,5-diphenyltetrazolium bromide) colorimetric assay (Sigma-Aldrich Chemie, Steinheim, Germany) was performed. A2780 cells were seeded in 96-well culture plates at a density of 2 × 10^3^ cells per well, and A2780cis cells at a density of 2.5 × 10^3^ cells per well. After 24 h of incubation, the tested compounds were added either alone or in combination. The chalcone analog 1C was added at concentrations of 5, 10, 20, 30, 40, and 50 μmol/L. N-acetylcysteine (NAC) (Merck, Darmstadt, Germany) was dissolved in purified water and added at concentrations of 0.1, 0.2, 0.3, 0.5, 1.0, 2.0, 2.5, and 3.0 mmol/L. The cells were then incubated for 12, 24, and 48 h. Subsequently, 10 μL of MTT (5 mg/mL) was added to each well. After an additional 1.5–2 h of incubation at 37 °C, 100 μL of 10% sodium dodecyl sulfate (SDS) was added to each well. Following a further 24-h incubation, during which formazan crystals dissolved, the metabolic activity of the cells was evaluated by measuring absorbance at a wavelength of 540 nm using a Cytation™ 3 Cell Imaging Multi-Mode Reader (Biotek, Winooski, VT, USA). The IC_50_ value was calculated from values obtained from three independent analyses using a nonlinear regression method.

### 4.4. Flow Cytometry Analyses

For the flow cytometry analysis purposes, A2780 and A2780cis cells were seeded in Petri dishes (6 cm) at densities of 2 × 10^5^ and 2.5 × 10^5^ respectively. After 24 h of incubation in a growth medium, cells were treated with 1C alone at a concentration of 7 μmol/L for A2780cis and 9 μmol/L for A2780, NAC alone at a concentration of 2.5 mmol/L and in combination with 1C at the concentrations mentioned above. The antioxidant compound NAC was added as a pre-treatment 30 min before the 1C addition. Exposure to tested compounds lasted for 12, 24, and 48 h. Cells were collected using trypsin, washed and resuspended in phosphate-buffered saline (PBS), and aliquoted into cytometric tubes for specific staining and subsequent analysis (see Section 4.4.1, Section 4.4.2, Section 4.4.3, Section 4.4.4, Section 4.4.5 and Section 4.4.6). After each staining cells were washed with PBS one more time. All cytometry analyses focused on fluorescence alteration were conducted using BD FACSCalibur flow cytometer (BD Biosciences, San Jose, CA, USA). A minimum of 1 × 10^4^ events were analyzed per assessment. An unstained control, an untreated control, and a DMSO control were used for each analysis. Each analysis was performed in three independent repetitions. Data obtained from analyses were evaluated using FlowJo software v.10 (BD Biosciences, San Jose, CA, USA).

#### 4.4.1. Apoptosis Analysis

Flow cytometric analysis focused on apoptosis was performed using Annexin V/PI staining. Cells were resuspended in 100 μL of PBS and Annexin V–Alexa Fluor^®^ 647 solution was added (1:300, Thermo Scientific, Rockford, IL, USA) according to the manufacturer’s instructions. After 15 min at room temperature in the dark and washing with PBS, 1 μL of propidium iodide (PI) (0.025 mg/mL, Sigma Aldrich, St. Louis, MO, USA) was added to the tubes. Data analysis used the FL-2 (585/42) vs. FL-4 (661/16) channels.

#### 4.4.2. Analysis of Mitochondrial Membrane Potential Changes (MMP)

To determine changes in MMP, cells were treated with tetramethylrhodamine ethyl ester (TMRE; Molecular Probes, Eugene, OR, USA) for 30 min at room temperature in the dark. The final concentration of TMRE was 0.1 μmol/L. Analysis was performed using the FL-2 (585/42) channel.

#### 4.4.3. Cell Cycle Analysis

For the cell cycle changes analysis, treated cells were collected after 12, 24, and 48 h, washed with PBS, fixed in 70% cold ethanol, and stored at −20 °C for at least 24 h. Before analysis, the cells were washed two times and resuspended in staining solution containing 0.1% Triton X-100 in PBS, ribonuclease A (0.5 mg/mL), and propidium iodide (0.025 mg/mL). Incubation at room temperature in the dark lasted for 30 min. Then the flow cytometry analysis was conducted using the FL-2 (585/42) channel.

#### 4.4.4. ROS Production Analysis

All samples except the negative control were stained with dihydrorhodamine 123 (DHR123) at a final concentration of 200 nmol/mL. Cells were incubated for 15 min at room temperature without access to light. After washing (1x PBS), fluorescence alterations were assessed.

#### 4.4.5. Lipid Peroxidation Analysis

To analyze lipid peroxidation in treated cells, BODIPY™ 581/591 C11 (Sigma-Aldrich, St. Louis, MO, USA) staining was used. The staining solution was added at a final concentration of 1 mmol/L for 15 min at room temperature in the dark. After washing with PBS, the acquisition was performed.

#### 4.4.6. Mitochondrial Superoxide Anion Production Analysis 

For the analysis of superoxide anion production in treated cells, staining was performed using MitoSOX™ Red Mitochondrial Superoxide Indicator (Thermo Scientific, Rockford, IL, USA) at a final concentration of 5 μmol/L. After 15 min at room temperature in the dark, the cells were washed, and the analysis was carried out.

### 4.5. Western Blot Analyses

For protein analysis, tested A2780 and A2780cis cells were seeded in Petri dishes (10 cm) in density 5 × 10^5^ per dish. After 24 h of incubation cells were treated with compound 1C (7 μmol/L for A2780cis and 9 μmol/L for A2780), NAC (2.5 μmol/L), and a combination of 1C+NAC. NAC used as s pretreatment was added 30 min before 1C. After 12, 24, and 48 h of exposure to tested compounds, cells were collected and protein lysates were prepared using Laemmli lysis buffer. After adding buffer consisting of 1 mol/L Tris/HCl (pH 6.8), glycerol, 20% SDS (sodium dodecyl sulfate), deionized H2O and phosphatase and protease inhibitors (Sigma-Aldrich, St. Louis, MO, USA) sonification was performed. The concentrations of cell proteins were detected by using Pierce^®^ BCA Protein Assay Kit (Thermo Scientific, Rockford, IL, USA) and by an automated CytationTM 3 Cell Imaging Multi-Mode Reader (Biotek, Winooski, VT, USA) at a wavelength of 570 nm. Proteins (30 μg per well) were loaded and electroporated using 12% SDS-PAA gel at 115 V for 3h and subsequently transferred to polyvinylidene difluoride (PVDF) membrane using the iBlotTM 2 Dry Blotting System (Invitrogen, Carlsbad, CA, USA). To prevent non-specific bindings, membranes with transferred proteins were blocked in 5% BSA (bovine serum albumin; SERVA, Heidelberg, Germany) dissolved in TBS-Tween (pH 7.4) for 1 h at room temperature. Membranes were incubated with primary antibodies diluted (Table 1) according to the manufacturer’s instructions in 5% BSA or 5% non-fat dry milk in TBS-Tween (pH7.4) (Cell Signaling Technology^®^, Danvers, MA, USA) for 24 h at 4 °C and washed in TBS-Tween (3 × 5 min). Depending on the primary antibody, membranes were incubated with horseradish peroxidase (HRP) conjugated anti-mouse or anti-rabbit secondary antibody for 1 h at room temperature. Afterward, washing was performed in TBS-Tween (3 × 5 min) and expression of proteins was detected using iBright TM FL1500 Imaging System with chemiluminescent ECL substrate (Thermo Scientific, Rockford, IL, USA). A densitometric analysis was conducted using the Image StudioTM Lite Software (LI-COR Biosciences, Lincoln, NE, USA, https://www.licor.com/bio/image-studio/, accecssed on 5 July 2024). Evaluation of the samples was performed in comparison with β-actin. All WB analyses were performed in three independent repetitions.

### 4.6. Statistical Analysis

The presented results are expressed as mean ± standard deviation (SD). Mean and standard deviations were calculated from a minimum of three independent experiments repetitions. Statistical analysis describing the significance between individual groups was conducted using a one-way analysis of variance (ANOVA) followed by the Bonferroni multiple comparison test. In this article, values *p* < 0.05 were considered as statistically significant, * indicates *p* < 0.05, ** *p* < 0.01, *** *p* < 0.001 vs. DMSO treated negative control; # *p* < 0.05, ## *p* < 0.01, ### *p* < 0.001 vs. 1C treated cells.

## 5. Conclusions

Our results demonstrated that the antiproliferative effects of the chalcone 1C on both sensitive and resistant ovarian cancer cells are closely associated with the induction of oxidative stress, as directly evidenced by increased production of ROS, superoxide, or elevated lipid peroxidation, or indirectly by the use of NAC. The 1C-induced decrease in cell viability, G2/M cell cycle arrest, and apoptosis were significantly attenuated by combination with NAC, suggesting the involvement of oxidative stress in the antiproliferative effect of this synthetic chalcone analog. Furthermore, surprising effects were observed regarding the influence of 1C on Nrf2 levels between sensitive and resistant ovarian cancer cells. It is generally accepted that Nrf2 signaling is activated as a result of oxidative stress. In our experiments, oxidative stress was most pronounced after 48 h of incubation. We expected that the chalcone 1C would suppress Nrf2 signaling, resulting in cell death. This outcome was observed in the sensitive cell line. On the other hand, in resistant cells, Nrf2 levels significantly increased after 48 h of incubation. We believe this is indicative of the resistant cells’ ability to combat adverse environments and reduce the risk of death. However, Further experiments will be necessary to provide evidence for this hypothesis.

## Figures and Tables

**Table 1:** List of primary and secondary Western blot antibodies.

Primary Antibody	Mr (Kda)	Origin	Dilution	Catologue No.	Manufacturer
β-Actin (8H10D10)	45	mouse	1:1000	#3700	Cell Signaling Technology^®^, Danvers, MA, USA
Phospho/Akt (Ser473) (D9E) XP^®^	60	rabbit		#4060	
Phospho-Bad (Ser112) (40A9)	23	rabbit		#5284	
Phospho-p44/42 MAPK (Erk1/2) (Thr202/Tyr204) (D13.14.4E) XP^®^	42/44	rabbit		#4370	
Phospho-Histone H2A.X (Ser139) (20E3)	15	rabbit		#9718	
Phospho-Rb (Ser807/811) (D20B12) XP^®^	110	rabbit		#8516	
p21 Waf1/Cip1 (12D1)	21	rabbit		#2947	
PARP (46D11)	116/89	rabbit		#9532	
PCNA (D3H8P) XP^®^ Rabbit mAb	36	rabbit		#13110	
NRF2 (D1Z9C) XP^®^	97–100	rabbit		#12721	
NF-κB1 p105/p50 (D7H5M)	105/50	rabbit		#12540	
NF-κB p65 (D14E12)	65	rabbit		#8242	
Secondary antibody					
anti-rabbit-IgG HRP linked	-	goat		#7074	
anti-mouse-IgG HRP linked	-	goat		#7075	

## Data Availability

Data are contained within the article and Supplementary Materials.

## Supplementary Materials

The following supporting information can be downloaded at: https://www.mdpi.com/article/10.3390/ijms25147541/s1, Table S1: Percentage distribution of A2780 cell populations after 12, 24 and 48 h treatment with 1C, NAC and NAC/1C based on Annexin V/PI staining; Table S2: Percentage distribution of A2780cis cell populations after 12, 24 and 48 h treatment with 1C, NAC and NAC/1C based on Annexin V/PI staining; Table S3: Percentage distribution of A2780 (left) and A2780cis (right) cell populations after 12, 24 and 48 h treatment with 1C, NAC and NAC/1C based on flow cytometry cell cycle analysis.

## References

[1] Bray F., Laversanne M., Sung H., Ferlay J., Siegel R.L., Soerjomataram I., Jemal A. (2024). Global cancer statistics 2022: GLOBOCAN estimates of incidence and mortality worldwide for 36 cancers in 185 countries. CA Cancer J. Clin..

[2] Kuroki L., Guntupalli S.R. (2020). Treatment of epithelial ovarian cancer. BMJ.

[3] Moore K., Colombo N., Scambia G., Kim B.G., Oaknin A., Friedlander M., Lisyanskaya A., Floquet A., Leary A., Sonke G.S. (2018). Maintenance Olaparib in Patients with Newly Diagnosed Advanced Ovarian Cancer. N. Engl. J. Med..

[4] Ray-Coquard I., Pautier P., Pignata S., Perol D., Gonzalez-Martin A., Berger R., Fujiwara K., Vergote I., Colombo N., Maenpaa J. (2019). Olaparib plus Bevacizumab as First-Line Maintenance in Ovarian Cancer. N. Engl. J. Med..

[5] Majd F.S., Talebi S.S., Ahmad Abadi A.N., Poorolajal J., Dastan D. (2022). Efficacy of a standardized herbal product from *Pistacia atlantica* subsp. Kurdica in type 2 diabetic patients with hyperlipidemia: A triple-blind randomized clinical trial. Complement. Ther. Clin. Pract..

[6] Hajiluian G., Karegar S.J., Shidfar F., Aryaeian N., Salehi M., Lotfi T., Farhangnia P., Heshmati J., Delbandi A.A. (2023). The effects of Ellagic acid supplementation on neurotrophic, inflammation, and oxidative stress factors, and indoleamine 2,3-dioxygenase gene expression in multiple sclerosis patients with mild to moderate depressive symptoms: A randomized, triple-blind, placebo-controlled trial. Phytomedicine.

[7] Kubatka P., Mazurakova A., Koklesova L., Kuruc T., Samec M., Kajo K., Kotorova K., Adamkov M., Smejkal K., Svajdlenka E. (2024). *Salvia officinalis* L. exerts oncostatic effects in rodent and in vitro models of breast carcinoma. Front. Pharmacol..

[8] Gao F., Huang G., Xiao J. (2020). Chalcone hybrids as potential anticancer agents: Current development, mechanism of action, and structure-activity relationship. Med. Res. Rev..

[9] Usui-Kawanishi F., Kani K., Karasawa T., Honda H., Takayama N., Takahashi M., Takatsu K., Nagai Y. (2024). Isoliquiritigenin inhibits NLRP3 inflammasome activation with CAPS mutations by suppressing caspase-1 activation and mutated NLRP3 aggregation. Genes Cells.

[10] Qian W., Lu J., Gao C., Liu Q., Yao W., Wang T., Wang X., Wang Z. (2024). Isobavachalcone exhibits antifungal and antibiofilm effects against *C. albicans* by disrupting cell wall/membrane integrity and inducing apoptosis and autophagy. Front. Cell Infect. Microbiol..

[11] Uddin J., Ali Shah S.W., Zahoor M., Ullah R., Alotaibi A. (2023). Chalcones: The flavonoid derivatives synthesis, characterization, their antioxidant and in vitro/in vivo antidiabetic potentials. Heliyon.

[12] Lin J.H., Yang K.T., Ting P.C., Lee W.S., Lin D.J., Chang J.C. (2023). Licochalcone a improves cardiac functions after ischemia-reperfusion via reduction of ferroptosis in rats. Eur. J. Pharmacol..

[13] Pei Z., Wu M., Yu H., Long G., Gui Z., Li X., Chen H., Jia Z., Xia W. (2022). Isoliquiritin Ameliorates Cisplatin-Induced Renal Proximal Tubular Cell Injury by Antagonizing Apoptosis, Oxidative Stress and Inflammation. Front. Med..

[14] Song L., Mino M., Yamak J., Nguyen V., Lopez D., Pham V., Fazelpour A., Le V., Fu D., Tippin M. (2022). Flavokawain A Reduces Tumor-Initiating Properties and Stemness of Prostate Cancer. Front. Oncol..

[15] Liu Z., Song L., Xie J., Simoneau A.R., Uchio E., Zi X. (2022). Chemoprevention of Urothelial Cell Carcinoma Tumorigenesis by Dietary Flavokawain A in UPII-Mutant Ha-ras Transgenic Mice. Pharmaceutics.

[16] de Freitas K.S., Squarisi I.S., Acesio N.O., Nicolella H.D., Ozelin S.D., Reis Santos de Melo M., Guissone A.P.P., Fernandes G., Silva L.M., da Silva Filho A.A. (2020). Licochalcone A, a licorice flavonoid: Antioxidant, cytotoxic, genotoxic, and chemopreventive potential. J. Toxicol. Environ. Health A.

[17] James S., Aparna J.S., Babu A., Paul A.M., Lankadasari M.B., Athira S.R., Kumar S.S., Vijayan Y., Namitha N.N., Mohammed S. (2021). Cardamonin Attenuates Experimental Colitis and Associated Colorectal Cancer. Biomolecules.

[18] Chang C.T., Hseu Y.C., Thiyagarajan V., Lin K.Y., Way T.D., Korivi M., Liao J.W., Yang H.L. (2017). Chalcone flavokawain B induces autophagic-cell death via reactive oxygen species-mediated signaling pathways in human gastric carcinoma and suppresses tumor growth in nude mice. Arch. Toxicol..

[19] Jin H., Seo G.S., Lee S.H. (2018). Isoliquiritigenin-mediated p62/SQSTM1 induction regulates apoptotic potential through attenuation of caspase-8 activation in colorectal cancer cells. Eur. J. Pharmacol..

[20] Liu H., Zhang L., Li G., Gao Z. (2020). Xanthohumol protects against Azoxymethane-induced colorectal cancer in Sprague-Dawley rats. Environ. Toxicol..

[21] Michalkova R., Mirossay L., Kello M., Mojzisova G., Baloghova J., Podracka A., Mojzis J. (2023). Anticancer Potential of Natural Chalcones: In Vitro and In Vivo Evidence. Int. J. Mol. Sci..

[22] Michalkova R., Mirossay L., Gazdova M., Kello M., Mojzis J. (2021). Molecular Mechanisms of Antiproliferative Effects of Natural Chalcones. Cancers.

[23] Xiao Q., Zhong B., Hou Y., Wang M., Guo B., Lin L., Zhou Y., Chen X. (2023). Fighting cancer by triggering non-canonical mitochondrial permeability transition-driven necrosis through reactive oxygen species induction. Free Radic. Biol. Med..

[24] WalyEldeen A.A., El-Shorbagy H.M., Hassaneen H.M., Abdelhamid I.A., Sabet S., Ibrahim S.A. (2022). [1,2,4] Triazolo [3,4-a]isoquinoline chalcone derivative exhibits anticancer activity via induction of oxidative stress, DNA damage, and apoptosis in Ehrlich solid carcinoma-bearing mice. Naunyn Schmiedebergs Arch. Pharmacol..

[25] Maciejewska N., Olszewski M., Jurasz J., Serocki M., Dzierzynska M., Cekala K., Wieczerzak E., Baginski M. (2022). Novel chalcone-derived pyrazoles as potential therapeutic agents for the treatment of non-small cell lung cancer. Sci. Rep..

[26] Panieri E., Pinho S.A., Afonso G.J.M., Oliveira P.J., Cunha-Oliveira T., Saso L. (2022). NRF2 and Mitochondrial Function in Cancer and Cancer Stem Cells. Cells.

[27] Tonelli C., Chio I.I.C., Tuveson D.A. (2018). Transcriptional Regulation by Nrf2. Antioxid. Redox Signal..

[28] Saha S., Buttari B., Panieri E., Profumo E., Saso L. (2020). An Overview of Nrf2 Signaling Pathway and Its Role in Inflammation. Molecules.

[29] Panieri E., Buha A., Telkoparan-Akillilar P., Cevik D., Kouretas D., Veskoukis A., Skaperda Z., Tsatsakis A., Wallace D., Suzen S. (2020). Potential Applications of NRF2 Modulators in Cancer Therapy. Antioxidants.

[30] Panieri E., Saso L. (2021). Inhibition of the NRF2/KEAP1 Axis: A Promising Therapeutic Strategy to Alter Redox Balance of Cancer Cells. Antioxid. Redox Signal..

[31] Liu P., Pan Q. (2022). Butein Inhibits Oxidative Stress Injury in Rats with Chronic Heart Failure via ERK/Nrf2 Signaling. Cardiovasc. Ther..

[32] Al-Qahtani W.H., Alshammari G.M., Ajarem J.S., Al-Zahrani A.Y., Alzuwaydi A., Eid R., Yahya M.A. (2022). Isoliquiritigenin prevents Doxorubicin-induced hepatic damage in rats by upregulating and activating SIRT1. Biomed. Pharmacother..

[33] Qi W., Boliang W., Xiaoxi T., Guoqiang F., Jianbo X., Gang W. (2020). Cardamonin protects against doxorubicin-induced cardiotoxicity in mice by restraining oxidative stress and inflammation associated with Nrf2 signaling. Biomed. Pharmacother..

[34] Gallorini M., Carradori S., Resende D., Saso L., Ricci A., Palmeira A., Cataldi A., Pinto M., Sousa E. (2022). Natural and Synthetic Xanthone Derivatives Counteract Oxidative Stress via Nrf2 Modulation in Inflamed Human Macrophages. Int. J. Mol. Sci..

[35] Laphanuwat P., Kongpetch S., Senggunprai L., Prawan A., Kukongviriyapan V. (2022). Licochalcone A Induces Cholangiocarcinoma Cell Death Via Suppression of Nrf2 and NF-kappaB Signaling Pathways. Asian Pac. J. Cancer Prev..

[36] Jin J., Qiu S., Wang P., Liang X., Huang F., Wu H., Zhang B., Zhang W., Tian X., Xu R. (2019). Cardamonin inhibits breast cancer growth by repressing HIF-1alpha-dependent metabolic reprogramming. J. Exp. Clin. Cancer Res..

[37] Lim J., Lee S.H., Cho S., Lee I.S., Kang B.Y., Choi H.J. (2013). 4-methoxychalcone enhances cisplatin-induced oxidative stress and cytotoxicity by inhibiting the Nrf2/ARE-mediated defense mechanism in A549 lung cancer cells. Mol. Cells.

[38] Takac P., Kello M., Pilatova M.B., Kudlickova Z., Vilkova M., Slepcikova P., Petik P., Mojzis J. (2018). New chalcone derivative exhibits antiproliferative potential by inducing G2/M cell cycle arrest, mitochondrial-mediated apoptosis and modulation of MAPK signalling pathway. Chem. Biol. Interact..

[39] Takac P., Kello M., Vilkova M., Vaskova J., Michalkova R., Mojzisova G., Mojzis J. (2020). Antiproliferative Effect of Acridine Chalcone Is Mediated by Induction of Oxidative Stress. Biomolecules.

[40] Gazdova M., Michalkova R., Kello M., Vilkova M., Kudlickova Z., Baloghova J., Mirossay L., Mojzis J. (2022). Chalcone-Acridine Hybrid Suppresses Melanoma Cell Progression via G2/M Cell Cycle Arrest, DNA Damage, Apoptosis, and Modulation of MAP Kinases Activity. Int. J. Mol. Sci..

[41] Yang L., Xie H.J., Li Y.Y., Wang X., Liu X.X., Mai J. (2022). Molecular mechanisms of platinum-based chemotherapy resistance in ovarian cancer (Review). Oncol. Rep..

[42] Song M., Cui M., Liu K. (2022). Therapeutic strategies to overcome cisplatin resistance in ovarian cancer. Eur. J. Med. Chem..

[43] Chen M., Gowd V., Wang M., Chen F., Cheng K.W. (2021). The apple dihydrochalcone phloretin suppresses growth and improves chemosensitivity of breast cancer cells via inhibition of cytoprotective autophagy. Food Funct..

[44] Hou G., Yuan X., Li Y., Hou G., Liu X. (2020). Cardamonin, a natural chalcone, reduces 5-fluorouracil resistance of gastric cancer cells through targeting Wnt/beta-catenin signal pathway. Investig. New Drugs.

[45] Wu C.P., Lusvarghi S., Hsiao S.H., Liu T.C., Li Y.Q., Huang Y.H., Hung T.H., Ambudkar S.V. (2020). Licochalcone A Selectively Resensitizes ABCG2-Overexpressing Multidrug-Resistant Cancer Cells to Chemotherapeutic Drugs. J. Nat. Prod..

[46] Wen D., Peng Y., Lin F., Singh R.K., Mahato R.I. (2017). Micellar Delivery of miR-34a Modulator Rubone and Paclitaxel in Resistant Prostate Cancer. Cancer Res..

[47] Zigova M., Miskufova V., Budovska M., Michalkova R., Mojzis J. (2024). Exploring the Antiproliferative and Modulatory Effects of 1-Methoxyisobrassinin on Ovarian Cancer Cells: Insights into Cell Cycle Regulation, Apoptosis, Autophagy, and Its Interactions with NAC. Molecules.

[48] Jung E., Koh D., Lim Y., Shin S.Y., Lee Y.H. (2020). Overcoming multidrug resistance by activating unfolded protein response of the endoplasmic reticulum in cisplatin-resistant A2780/CisR ovarian cancer cells. BMB Rep..

[49] Gil H.N., Jung E., Koh D., Lim Y., Lee Y.H., Shin S.Y. (2019). A synthetic chalcone derivative, 2-hydroxy-3′,5,5′-trimethoxychalcone (DK-139), triggers reactive oxygen species-induced apoptosis independently of p53 in A549 lung cancer cells. Chem. Biol. Interact..

[50] He H., Wang C., Liu G., Ma H., Jiang M., Li P., Lu Q., Li L., Qi H. (2021). Isobavachalcone inhibits acute myeloid leukemia: Potential role for ROS-dependent mitochondrial apoptosis and differentiation. Phytother. Res..

[51] Hseu Y.C., Chiang Y.C., Vudhya Gowrisankar Y., Lin K.Y., Huang S.T., Shrestha S., Chang G.R., Yang H.L. (2020). The In Vitro and In Vivo Anticancer Properties of Chalcone Flavokawain B through Induction of ROS-Mediated Apoptotic and Autophagic Cell Death in Human Melanoma Cells. Cancers.

[52] Zhang S., Li T., Zhang L., Wang X., Dong H., Li L., Fu D., Li Y., Zi X., Liu H.M. (2017). A novel chalcone derivative S17 induces apoptosis through ROS dependent DR5 up-regulation in gastric cancer cells. Sci. Rep..

[53] Sahoo B.M., Banik B.K., Borah P., Jain A. (2022). Reactive Oxygen Species (ROS): Key Components in Cancer Therapies. Anticancer Agents Med. Chem..

[54] Inoue T., Suzuki-Karasaki Y. (2013). Mitochondrial superoxide mediates mitochondrial and endoplasmic reticulum dysfunctions in TRAIL-induced apoptosis in Jurkat cells. Free Radic. Biol. Med..

[55] Farooqi A.A., Li K.T., Fayyaz S., Chang Y.T., Ismail M., Liaw C.C., Yuan S.S., Tang J.Y., Chang H.W. (2015). Anticancer drugs for the modulation of endoplasmic reticulum stress and oxidative stress. Tumour Biol..

[56] Santarsiero A., Pappalardo I., Rosa G.M., Pisano I., Superchi S., Convertini P., Todisco S., Scafato P., Infantino V. (2022). Mitochondrial Role in Intrinsic Apoptosis Induced by a New Synthesized Chalcone in Hepatocellular Carcinoma Cells. Biomedicines.

[57] Travnicek Z., Vanco J., Belza J., Zoppellaro G., Dvorak Z. (2024). Dinuclear copper(II) complexes with a bridging bis(chalcone) ligand reveal considerable in vitro cytotoxicity on human cancer cells and enhanced selectivity. J. Inorg. Biochem..

[58] Wang Z., Li W., Wang X., Zhu Q., Liu L., Qiu S., Zou L., Liu K., Li G., Miao H. (2023). Isoliquiritigenin induces HMOX1 and GPX4-mediated ferroptosis in gallbladder cancer cells. Chin. Med. J..

[59] Zhang C., Ding Q., Xia Z., Wang H., Jiang F., Lu Y. (2023). Novel Chalcone-Phenazine Hybrids Induced Ferroptosis in U87-MG Cells through Activating Ferritinophagy. Chem. Biodivers..

[60] Su L.J., Zhang J.H., Gomez H., Murugan R., Hong X., Xu D., Jiang F., Peng Z.Y. (2019). Reactive Oxygen Species-Induced Lipid Peroxidation in Apoptosis, Autophagy, and Ferroptosis. Oxid. Med. Cell. Longev..

[61] Iqbal M.J., Kabeer A., Abbas Z., Siddiqui H.A., Calina D., Sharifi-Rad J., Cho W.C. (2024). Interplay of oxidative stress, cellular communication and signaling pathways in cancer. Cell Commun. Signal..

[62] Patterson J.C., Joughin B.A., van de Kooij B., Lim D.C., Lauffenburger D.A., Yaffe M.B. (2019). ROS and Oxidative Stress Are Elevated in Mitosis during Asynchronous Cell Cycle Progression and Are Exacerbated by Mitotic Arrest. Cell Syst..

[63] Li K., Zhao S., Long J., Su J., Wu L., Tao J., Zhou J., Zhang J., Chen X., Peng C. (2020). A novel chalcone derivative has antitumor activity in melanoma by inducing DNA damage through the upregulation of ROS products. Cancer Cell Int..

[64] Toettcher J., Dubitzky W., Wolkenhauer O., Cho K.-H., Yokota H. (2013). Cell Cycle Arrest after DNA Damage. Encyclopedia of Systems Biology.

[65] Michalkova R., Kello M., Kudlickova Z., Gazdova M., Mirossay L., Mojzisova G., Mojzis J. (2022). Programmed Cell Death Alterations Mediated by Synthetic Indole Chalcone Resulted in Cell Cycle Arrest, DNA Damage, Apoptosis and Signaling Pathway Modulations in Breast Cancer Model. Pharmaceutics.

[66] Nevins J.R. (2001). The Rb/E2F pathway and cancer. Hum. Mol. Genet..

[67] Boehm E.M., Gildenberg M.S., Washington M.T. (2016). The Many Roles of PCNA in Eukaryotic DNA Replication. Enzymes.

[68] Zhang Y., Yang J., Wen Z., Chen X., Yu J., Yuan D., Xu B., Luo H., Zhu J. (2020). A novel 3′,5′-diprenylated chalcone induces concurrent apoptosis and GSDME-dependent pyroptosis through activating PKCdelta/JNK signal in prostate cancer. Aging.

[69] Huang Z., Wu Q., Wang Z. (2020). Anti-tumor effects of isoliquiritigenin in Bcl-2/Bax and PCNA expression of T24 human bladder cancer cells. Arch. Med. Sci..

[70] Su D., Lv C. (2021). Hydroxysafflor yellow A inhibits the proliferation, migration, and invasion of colorectal cancer cells through the PPARgamma/PTEN/Akt signaling pathway. Bioengineered.

[71] Chen J. (2016). The Cell-Cycle Arrest and Apoptotic Functions of p53 in Tumor Initiation and Progression. Cold Spring Harb. Perspect. Med..

[72] Qi Z., Liu M., Liu Y., Zhang M., Yang G. (2014). Tetramethoxychalcone, a chalcone derivative, suppresses proliferation, blocks cell cycle progression, and induces apoptosis of human ovarian cancer cells. PLoS ONE.

[73] Hseu Y.C., Chiang Y.C., Vudhya Gowrisankar Y., Lin K.Y., Huang S.T., Shrestha S., Chang G.R., Yang H.L. (2021). Correction: Hseu, Y.-C. et al. The In Vitro and In Vivo Anticancer Properties of Chalcone Flavokawain B through Induction of ROS-Mediated Apoptotic and Autophagic Cell Death in Human Melanoma Cells. Cancers **2020**, *12*, 2936. Cancers.

[74] Tait S.W., Green D.R. (2013). Mitochondrial regulation of cell death. Cold Spring Harb. Perspect. Biol..

[75] Kwak A.W., Lee M.J., Lee M.H., Yoon G., Cho S.S., Chae J.I., Shim J.H. (2021). The 3-deoxysappanchalcone induces ROS-mediated apoptosis and cell cycle arrest via JNK/p38 MAPKs signaling pathway in human esophageal cancer cells. Phytomedicine.

[76] Kwak A.W., Choi J.S., Lee M.H., Oh H.N., Cho S.S., Yoon G., Liu K., Chae J.I., Shim J.H. (2019). Retrochalcone Echinatin Triggers Apoptosis of Esophageal Squamous Cell Carcinoma via ROS- and ER Stress-Mediated Signaling Pathways. Molecules.

[77] Wang L.H., Li H.H., Li M., Wang S., Jiang X.R., Li Y., Ping G.F., Cao Q., Liu X., Fang W.H. (2015). SL4, a chalcone-based compound, induces apoptosis in human cancer cells by activation of the ROS/MAPK signalling pathway. Cell Prolif..

[78] Hseu Y.C., Lee M.S., Wu C.R., Cho H.J., Lin K.Y., Lai G.H., Wang S.Y., Kuo Y.H., Kumar K.J., Yang H.L. (2012). The chalcone flavokawain B induces G2/M cell-cycle arrest and apoptosis in human oral carcinoma HSC-3 cells through the intracellular ROS generation and downregulation of the Akt/p38 MAPK signaling pathway. J. Agric. Food Chem..

[79] Gasparri M.L., Bardhi E., Ruscito I., Papadia A., Farooqi A.A., Marchetti C., Bogani G., Ceccacci I., Mueller M.D., Benedetti Panici P. (2017). PI3K/AKT/mTOR Pathway in Ovarian Cancer Treatment: Are We on the Right Track?. Geburtshilfe Frauenheilkd..

[80] Zhao Y., Hu X., Liu Y., Dong S., Wen Z., He W., Zhang S., Huang Q., Shi M. (2017). ROS signaling under metabolic stress: Cross-talk between AMPK and AKT pathway. Mol. Cancer.

[81] Deeb D., Gao X., Jiang H., Arbab A.S., Dulchavsky S.A., Gautam S.C. (2010). Growth inhibitory and apoptosis-inducing effects of xanthohumol, a prenylated chalone present in hops, in human prostate cancer cells. Anticancer Res..

[82] Wani Z.A., Guru S.K., Rao A.V., Sharma S., Mahajan G., Behl A., Kumar A., Sharma P.R., Kamal A., Bhushan S. (2016). A novel quinazolinone chalcone derivative induces mitochondrial dependent apoptosis and inhibits PI3K/Akt/mTOR signaling pathway in human colon cancer HCT-116 cells. Food Chem. Toxicol..

[83] Navaei Z.N., Khalili-Tanha G., Zangouei A.S., Abbaszadegan M.R., Moghbeli M. (2021). PI3K/AKT signaling pathway as a critical regulator of Cisplatin response in tumor cells. Oncol. Res..

[84] Peng D.J., Wang J., Zhou J.Y., Wu G.S. (2010). Role of the Akt/mTOR survival pathway in cisplatin resistance in ovarian cancer cells. Biochem. Biophys. Res. Commun..

[85] Iida M., Harari P.M., Wheeler D.L., Toulany M. (2020). Targeting AKT/PKB to improve treatment outcomes for solid tumors. Mutat. Res..

[86] Lee S.O., Joo S.H., Lee J.Y., Kwak A.W., Kim K.T., Cho S.S., Yoon G., Choi Y.H., Park J.W., Shim J.H. (2024). Licochalcone C Inhibits the Growth of Human Colorectal Cancer HCT116 Cells Resistant to Oxaliplatin. Biomol. Ther..

[87] Wang J., Zhou J.Y., Wu G.S. (2007). ERK-dependent MKP-1-mediated cisplatin resistance in human ovarian cancer cells. Cancer Res..

[88] Li Y., Zhao M., Lin Y., Jiang X., Jin L., Ye P., Lu Y., Pei R., Jiang L. (2024). Licochalcone A induces mitochondria-dependent apoptosis and interacts with venetoclax in acute myeloid leukemia. Eur. J. Pharmacol..

[89] Lee J.Y., Lee S.O., Kwak A.W., Chae S.B., Cho S.S., Yoon G., Kim K.T., Choi Y.H., Lee M.H., Joo S.H. (2023). 3-Deoxysappanchalcone Inhibits Cell Growth of Gefitinib-Resistant Lung Cancer Cells by Simultaneous Targeting of EGFR and MET Kinases. Biomol. Ther..

[90] Jiang M., Zhou L.Y., Xu N., An Q. (2019). Hydroxysafflor yellow A inhibited lipopolysaccharide-induced non-small cell lung cancer cell proliferation, migration, and invasion by suppressing the PI3K/AKT/mTOR and ERK/MAPK signaling pathways. Thorac. Cancer.

[91] Ngo V., Duennwald M.L. (2022). Nrf2 and Oxidative Stress: A General Overview of Mechanisms and Implications in Human Disease. Antioxidants.

[92] de Freitas Silva M., Pruccoli L., Morroni F., Sita G., Seghetti F., Viegas C., Tarozzi A. (2018). The Keap1/Nrf2-ARE Pathway as a Pharmacological Target for Chalcones. Molecules.

[93] Bottoni L., Minetti A., Realini G., Pio E., Giustarini D., Rossi R., Rocchio C., Franci L., Salvini L., Catona O. (2024). NRF2 activation by cysteine as a survival mechanism for triple-negative breast cancer cells. Oncogene.

[94] Wang P., Jin J.M., Liang X.H., Yu M.Z., Yang C., Huang F., Wu H., Zhang B.B., Fei X.Y., Wang Z.T. (2022). Helichrysetin inhibits gastric cancer growth by targeting c-Myc/PDHK1 axis-mediated energy metabolism reprogramming. Acta Pharmacol. Sin..

[95] Ran H., Liu H., Wu P. (2021). Echinatin mitigates H_2_O_2_-induced oxidative damage and apoptosis in lens epithelial cells via the Nrf2/HO-1 pathway. Adv. Clin. Exp. Med..

[96] No J.H., Kim Y.B., Song Y.S. (2014). Targeting nrf2 signaling to combat chemoresistance. J. Cancer Prev..

[97] Verzella D., Pescatore A., Capece D., Vecchiotti D., Ursini M.V., Franzoso G., Alesse E., Zazzeroni F. (2020). Life, death, and autophagy in cancer: NF-kappaB turns up everywhere. Cell Death Dis..

[98] Pomerantz J.L., Baltimore D. (2002). Two pathways to NF-kappaB. Mol. Cell.

[99] Papierska K., Krajka-Kuzniak V., Kleszcz R., Stefanski T., Kurczab R., Kubicki M. (2022). The synthesis of novel thioderivative chalcones and their influence on NF-kappaB, STAT3 and NRF2 signaling pathways in colorectal cancer cells. Sci. Rep..

[100] Wang X., Liang Y., Zhang B., He L., Li W., Zhang W., Li C., Luo L., Umar T., Feng H. (2024). 2′-Hydroxychalcone Induces Autophagy and Apoptosis in Breast Cancer Cells via the Inhibition of the NF-kappaB Signaling Pathway: In Vitro and In Vivo Studies. Nutrients.

[101] Nourbakhsh M., Noori S., Aminzade Z., Bayanati M., Alemi M., Zarghi A. (2023). Attenuation of Inflammatory Responses in Breast and Ovarian Cancer Cells by a Novel Chalcone Derivative and Its Increased Potency by Curcumin. Mediat. Inflamm..

[102] Wang J.R., Luo Y.H., Piao X.J., Zhang Y., Feng Y.C., Li J.Q., Xu W.T., Zhang Y., Zhang T., Wang S.N. (2019). Mechanisms underlying isoliquiritigenin-induced apoptosis and cell cycle arrest via ROS-mediated MAPK/STAT3/NF-kappaB pathways in human hepatocellular carcinoma cells. Drug Dev. Res..

[103] Godwin P., Baird A.M., Heavey S., Barr M.P., O’Byrne K.J., Gately K. (2013). Targeting nuclear factor-kappa B to overcome resistance to chemotherapy. Front. Oncol..

