# Supplementary material for: The Induction of G2/M Phase Cell Cycle Arrest and Apoptosis by the Chalcone Derivative 1C in Sensitive and Resistant Ovarian Cancer Cells Is Associated with ROS Generation
Source: Int J Mol Sci. 2024 Jul 9;25(14):7541. doi: 10.3390/ijms25147541 (PMC11277160; doi:10.3390/ijms25147541)
Supplement: Supplementary file 1 [file ijms-25-07541-s001.zip › ijms-3082480-supplementary.pdf]

International Journal of *Molecular Sciences*

MDPI

Supplementary materials: The induction of G2/M phase cell cycle arrest and apoptosis by the chalcone derivative 1C in sensitive and resistant ovarian cancer cells is associated with ROS generation

Šimon Salanci [1], Mária Vilková [2], Lola Martinez [3], Ladislav Mirossay [1], Radka Michalková [1*] and Ján Mojžiš [1,*]

[1] Department of Pharmacology, Faculty of Medicine, Pavol Jozef Šafárik University, 040 01 Košice, Slovak Republic; simon.salanci@student.upjs.sk (S.S); ladislav.mirossay@upjs.sk (L.M.), radka.michalkova@upjs.sk (R.M); jan.mojzis@upjs.sk (J.M)

[2] Institute of Chemistry, Faculty of Science, Pavol Jozef Šafárik University, 040 01 Košice, Slovak Republic; maria.vilkova@upjs.sk (M.V)

[3] Flow Cytometry Unit, Biotechnology Programme, Spanish National Cancer Research Center (CNIO), Madrid, Spain; lmartinez@cnio.es (L.Ma.)

* Correspondence: jan.mojzis@upjs.sk (J.M)

Apoptosis analysis		A2780			
		An-/PI-	An+/PI-	An+/PI+	An-/PI+
12h	DMSO	89.80 ± 3.48	2.03 ± 1.29	0.74 ± 0.26	6.75 ± 1.49
	1C	80.65 ± 5.56	4,57 ± 4.82	1.83 ± 1.58	6.48 ± 2.28
	NAC	92.10 ± 3.41	2.71 ±1.66	1.28 ± 0.91	3.90 ± 1.15
	NAC/1C	92.00 ± 2.21	2.20 ± 1.18	1.00 ± 0.51	4.80 ± 1.04
24h	DMSO	93.23 ± 2.21	2.88 ± 2.01	0.90 ± 0.62	2.93 ± 0.34
	1C	78.05 ± 0.35 *	3.60 ± 2.01	4.03 ± 0.63 *	14.20 ± 0.10 ***
	NAC	93.23 ± 3.08 #	2.53 ± 1.59	1.07 ± 0.97	3.12 ± 0.91 ###
	NAC/1C	92.70 ± 0.22###	2.90 ± 1.73	3.10 ± 3.75	7.36 ± 4.04
48h	DMSO	95.03 ± 1.70	0.66 ± 0.34	0.57 ± 0.33	3,71 ± 1.06
	1C	57.86 ± 3.05 ***	15.60 ± 2.20 **	11.57 ± 2.44 **	11.57 ± 2.16 **
	NAC	91.40 ± 3.02 ##	1.69 ± 0.93 ##	1.20 ± 1.07 ##	5.73 ± 2.30 #
	NAC/1C	90.20 ± 6.44 #	1.77 ± 0.83 ##	1.83 ± 1.54 ##	6.20 ± 4,55

Table S1. Percentage distribution of A2780 cell populations after 12, 24 and 48 h treatment with 1C, NAC and NAC/1C based on Annexin V/PI staining. Data were obtained from 3 independent acquisitions and are presented as the mean ± standard deviation. Statistical significance: * $p<0.05$, **$p<0.01$, ***$p<0.001$ vs. control (DMSO) ; # $p<0.05$, ##$p<0.01$, ###$p<0.001$ vs. 1C.

Apoptosis analysis		A2780cis			
		An-/PI-	An+/PI-	An+/PI+	An-/PI+
12h	DMSO	93.33 ± 4.51	2.93 ± 1.65	1.70 ± 1.16	2.03 ± 2.26
	1C	84.07 ± 3.64	4.62 ± 3.08	3.80 ± 0.14 *	8.62 ± 2.35
	NAC	93.90 ± 1.70	2.10 ± 1.28	1.93 ± 1.18	2.00 ± 1.04 #
	NAC/1C	92.73 ± 3.43	2.90 ± 2.59	1.75 ± 0.32	2.57 ± 0.63 #
24h	DMSO	91.70 ± 2.50	1:93 ± 1.37	3.60 ± 0.30	2.70 ± 0.80
	1C	84.60 ± 3.00	3.31 ± 1.59	9.04 ± 2.97	3.11 ± 1.51
	NAC	94.35 ± 2.65	2.35 ± 1.75	1.50 ± 0.10 *	1.69 ± 0.72
	NAC/1C	89.90 ± 1.60	2.40 ± 1.70	3.70 ± 0.90	3.67 ± 0.43
48h	DMSO	94.05 ± 1.65	3.50 ± 1.40	1.87 ± 0.37	0.57 ± 0.13
	1C	49.85 ± 0.35**	25.45 ± 3.35 *	21.90 ± 3,60 *	2.71 ± 0.51
	NAC	92.35 ± 4.15 ##	3.70 ± 1.90 #	2.20 ± 1.20 #	1.70 ± 1.10
	NAC/1C	93.80 ± 2.50 ##	2.38 ± 0.82 #	1.76 ± 0.54 #	1.99 ± 1.12

Table S2. Percentage distribution of A2780cis cell populations after 12, 24 and 48 h treatment with 1C, NAC and NAC/1C based on Annexin V/PI staining. Data were obtained from 3 independent acquisitions and are presented as the mean ± standard deviation. Statistical significance: * p<0.05, **p<0.01, ***p<0.001 vs. control (DMSO) ; # p<0.05, ##p<0.01, ###p<0.001 vs. 1C.

Cell cycle analysis		A2780			A2780cis		
		G1	S	G2/M	G1	S	G2/M
12h	DMSO	45.03 ± 2.25	37.33 ± 3.23	18.30 ± 0.21	45.17 ± 3.56	39.97 ± 5.49	12.83 ± 2.08
	1C	43.20 ± 4.10	16.20 ± 5.10 *	39.40 ± 1.10 ***	30.75 ± 4.25 *	35.10 ± 4.90	31.75 ± 6.75 *
	NAC	47.80 ± 0.40	37.00 ± 1.30 #	14.00 ± 0.70 *#	45.15 ± 3.85	40.95 ± 5.15	12.40 ± 1.10
	NAC/1C	52.55 ± 1.05	33.10 ± 0.20	13.35 ± 0.85 *#	48.10 ± 2.30	38.20 ± 0.40	10.63 ± 1.88
24h	DMSO	48.30 ± 2,55	32.73 ± 2.49	17.43 ± 3.81	46.25 ± 0.45	40.50 ± 0.45	11.35 ± 0.75
	1C	37.83 ± 1.28 **	24.00 ± 11.58	37.65 ± 4.95 **	28.00 ± 0.80 **	34.20 ± 1.40 *	36.55 ± 3.45 **
	NAC	47.60 ± 3.16 #	36.93 ± 3.29	15.73 ± 1.53 ##	41.90 ± 4:70	45.15 ± 4.85	11.80 ± 0.20 ##
	NAC/1C	48.17 ± 5.67	35.70 ± 4.47	16.66 ± 1.40 ##	41.40 ± 4.40	46.20 ± 3.60	10.28 ± 1.02 ##
48h	DMSO	50.80 ± 1.96	35.53 ± 0.99	13.09 ± 1.98	49.30 ± 4.30	36.65 ± 4.75	11.85 ± 0.75
	1C	48.55 ± 1.35	19.25 ± 4.25 *	32.80 ± 2.40 **	44.35 ± 3.55 **	14.70 ± 1.00 **	39.15 ± 2.05
	NAC	55.50 ± 1.48 #	31.6 ± 1.95 #	13.27 ± 1.34 ##	45.95 ± 1.05	42.35 ± 2.35 ##	10.20 ± 2.80 #
	NAC/1C	54.63 ± 1.75 #	33.27 ± 2.25 #	12.03 ± 1.61 ##	53.30 ± 4.20	37.6 ± 5.00 #	7.13 ± 1.48 ##

Table S3. Percentage distribution of A2780 (left) and A2780cis (right) cell populations after 12, 24 and 48 h treatment with 1C, NAC and NAC/1C based on flow cytometry cell cycle analysis. Data were obtained from 3 independent acquisitions and are presented as the mean ± standard deviation. Statistical significance: * p<0.05, **p<0.01, ***p<0.001 vs. control (DMSO) ; # p<0.05, ##p<0.01, ###p<0.001 vs. 1C.
